# Two Birds One Stone: The Neuroprotective Effect of Antidiabetic Agents on Parkinson Disease—Focus on Sodium-Glucose Cotransporter 2 (SGLT2) Inhibitors

**DOI:** 10.3390/antiox10121935

**Published:** 2021-12-02

**Authors:** Kai-Jung Lin, Tzu-Jou Wang, Shang-Der Chen, Kai-Lieh Lin, Chia-Wei Liou, Min-Yu Lan, Yao-Chung Chuang, Jiin-Haur Chuang, Pei-Wen Wang, Jong-Jer Lee, Feng-Sheng Wang, Hung-Yu Lin, Tsu-Kung Lin

**Affiliations:** 1 Center for Mitochondrial Research and Medicine, Kaohsiung Chang Gung Memorial Hospital and Chang Gung University College of Medicine, Kaohsiung 83301, Taiwan; b101101092@tmu.edu.tw (K.-J.L.); tzujouw@gmail.com (T.-J.W.); jp1916@ms4.hinet.net (S.-D.C.); 100311039@gms.tcu.edu.tw (K.-L.L.); cwliou@ms22.hinet.net (C.-W.L.); myl@ksts.seed.net.tw (M.-Y.L.); jhchuang@cgmh.org.tw (J.-H.C.); wangpw@cgmh.org.tw (P.-W.W.); tojjlee@cgmh.org.tw (J.-J.L.); wangfs@ms33.hinet.net (F.-S.W.); linhungyu700218@gmail.com (H.-Y.L.); 2 Department of Family Medicine, National Taiwan University Hospital, Taipei 100225, Taiwan; 3 Department of Pediatric, Kaohsiung Chang Gung Memorial Hospital and Chang Gung University College of Medicine, Kaohsiung 83301, Taiwan; 4 Department of Neurology, Kaohsiung Chang Gung Memorial Hospital and Chang Gung University College of Medicine, Kaohsiung 83301, Taiwan; ycchuang@cgmh.org.tw; 5 Center of Parkinson’s Disease, Kaohsiung Chang Gung Memorial Hospital and Chang Gung University College of Medicine, Kaohsiung 83301, Taiwan; 6 Department of Anesthesiology, Kaohsiung Chang Gung Memorial Hospital and Chang Gung University College of Medicine, Kaohsiung 83301, Taiwan; 7 Department of Pediatric Surgery, Kaohsiung Chang Gung Memorial Hospital and Chang Gung University College of Medicine, Kaohsiung 83301, Taiwan; 8 Department of Metabolism, Kaohsiung Chang Gung Memorial Hospital and Chang Gung University College of Medicine, Kaohsiung 83301, Taiwan; 9 Department of Ophthalmology, Kaohsiung Chang Gung Memorial Hospital and Chang Gung University College of Medicine, Kaohsiung 83301, Taiwan; 10 Department of Medical Research, Kaohsiung Chang Gung Memorial Hospital, Kaohsiung 83301, Taiwan; 11 Research Assistant Center, Show Chwan Memorial Hospital, Changhua 500, Taiwan

**Keywords:** Parkinson’s disease, mitochondrial dysfunction, oxidative stress, SGLT2, antioxidative effects, diabetes, metabolic syndrome

## Abstract

Parkinson’s disease (PD) is the second most common neurodegenerative disease after Alzheimer’s disease affecting more than 1% of the population over 65 years old. The etiology of the disease is unknown and there are only symptomatic managements available with no known disease-modifying treatment. Aging, genes, and environmental factors contribute to PD development and key players involved in the pathophysiology of the disease include oxidative stress, mitochondrial dysfunction, autophagic–lysosomal imbalance, and neuroinflammation. Recent epidemiology studies have shown that type-2 diabetes (T2DM) not only increased the risk for PD, but also is associated with PD clinical severity. A higher rate of insulin resistance has been reported in PD patients and is suggested to be a pathologic driver in this disease. Oral diabetic drugs including sodium-glucose cotransporter 2 (SGLT2) inhibitors, glucagon-like peptide-1 (GLP-1) receptor agonists, and dipeptidyl peptidase-4 (DPP-4) inhibitors have been shown to provide neuroprotective effects in both PD patients and experimental models; additionally, antidiabetic drugs have been demonstrated to lower incidence rates of PD in DM patients. Among these, the most recently developed drugs, SGLT2 inhibitors may provide neuroprotective effects through improving mitochondrial function and antioxidative effects. In this article, we will discuss the involvement of mitochondrial-related oxidative stress in the development of PD and potential benefits provided by antidiabetic agents especially focusing on sglt2 inhibitors.

## 1. Introduction

Parkinson’s disease (PD) is the most common progressive neurodegenerative movement disorder with increasing prevalence among societies with a growing aging population [1]. Beyond the cardinal motor symptoms, tremor, rigidity, bradykinesia/akinesia, and postural instability, the life quality of PD patients is also compromised by non-motor features including cognitive impairment, hyposmia, sleep disorders, depression, constipation, and autonomic dysfunction [2,3]. Characteristic pathological hallmarks include the dopaminergic neuronal loss notably in the substantia nigra pars compacta and the accumulation of intraneuronal proteinaceous inclusions termed Lewy bodies which are enriched in α-synuclein protein aggregates [4]. As the disease progress, loss of dopaminergic neurons spread from the ventrolateral substantia nigra in the early-stage to become widespread to several different brain regions. Meanwhile, the Lewy pathology is observed initially in the olfactory system, then cholinergic, monoaminergic brainstem neurons, and to the limbic and neocortical brain regions with disease progression according to Braak’s pathological classification. Based on canonical understanding from modern neuroscience, effective symptomatic treatment is available with dopaminergic replacement therapy as the golden standard for PD. However, there is still no cure for the slowly progressive nature of this degenerative disease and a better understanding of the pathophysiology of PD may render clues for disease modification of this notorious disease. The exact etiology underlying PD is not certain but relative contributions of age, genes, environmental factors, and lifestyle are reported [5]. In the 1980s, 1-methyl-4-phenyl-1,2,3,6-tetrahydropyridine (MPTP), a mitochondrial complex I inhibitor, was found to induce parkinsonism in patients exposed to synthetic heroin contaminated with this neurotoxin [6]. Since then, the role of mitochondria in PD pathogenesis was highlighted. Supporting this, other pesticides, herbicides, and heavy metals known to increase PD risk based on epidemiology studies were also found to cause mitochondrial dysfunction [7,8,9]. In animal models of PD, neuronal death and neurobehavior deficits can be caused by mitochondrial dysfunction and oxidative stress which were linked to intracellular calcium balancing, redox homeostasis, the autophagic-lysosomal system, and mitochondria-dependent apoptosis [10,11]. Although most PD cases are sporadic in nature (~95%), approximately 3% of the sporadic and 30% of the familial PD cases are caused by monogenic mutations, functional studies of these involved PD susceptible genes provide crucial information of PD pathogenesis [12]. Pathogenic mutations leading to autosomal recessive forms of PD have been reported in PRKN (PARK2), PINK1 (PARK6), DJ-1 (PARK7), ATP13A2 (PARK9), and FBXO7 (PARK15), PLA2G6 (PARK14), DNAJC6 (PARK19a,b) SYNJ1 (PARK20), and VPS13C (PARK23) while the autosomal dominant PD genes involved include the SNCA (PARK1), LRRK2 (PARK8), VPS35 (PARK17), and CHCHD2 [13,14,15]. The above-mentioned genes and proteins they encode are all now known to be involved in the mitochondrial quality control system, mitophagy in particular, and the tightly related autophagic-lysosomal system [16,17]. Evidence for further involvement of mitochondrial processes in sporadic PD was suggested by Billingsley et al. that it is not only mitochondrial quality control and homeostasis, which contributes to PD risk but also other key mitochondrial processes, such as the function of mitochondrial ribosomes; this mirrors the biological complexity of mitochondrial disorders and a proportion of the “missing heritability” of sporadic PD (about 23%) can be explained by additive common genetic variation [18]. Participation of the autophagic-lysosomal system in PD is also reported clinically as a higher incidence of PD is observed in the lysosomal storage disorder Gaucher disease and the discovery of mutations in the glucocerebrosidase (GBA) gene in the development of idiopathic PD [19]. Mitochondria produce most of the intracellular energy adenosine triphosphate (ATP) from the electron transport chain (ETC) during the process of oxidative phosphorylation (OXPHOS) with reactive oxygen species (ROS) as byproducts. In redox homeostasis, intracellular antioxidative mechanisms balance the ROS produced in the mitochondria and do not induce cellular stress; however, under excessive ROS production or decreased antioxidative effects, oxidative stress occurs leading to oxidative damage of macromolecules such as DNA, proteins, and phospholipids.

In recent decades, an emerging body of evidence has connected PD and type 2 diabetes mellitus (T2DM) [20]. In cohort studies and a meta-analysis covering different ethnics including Caucasians, Asians, and Africans, pre-existent T2DM was reported not only to be associated with increased risk of developing PD, but also with faster progression rate and a more severe phenotype [21,22]. In addition, 50–80% of patients with PD have abnormal glucose tolerance which can be further exacerbated by levodopa therapy and the severity of hyperglycemia is related to nigrostriatal dopaminergic neurodegeneration in experimental animals [23,24]. The disruption of the insulin signaling can lead to insulin dysregulation, mitochondrial dysfunction, neuroinflammation, altered synaptic plasticity, and eventually neurodegeneration [25]. Therefore, there may be a shared common pathogenesis between PD and T2DM. Current investigation into the usage of established diabetes drugs in the management of PD is now under trial including sodium-glucose cotransporter 2 (SGLT2) inhibitors, glucagon-like peptide-1 receptor agonists (GLP-1RA), and dipeptidyl peptidase-4 (DPP-4) inhibitors. Among these, the recent star of T2DM control and cardiovascular diseases, the SGLT2 inhibitors, also called gliflozins, are a class of medications that inhibits reabsorption of glucose via the SGLT2 in the kidney and therefore lower blood sugar. Apart from blood sugar control, SGLT2 inhibitors have been shown to provide significant cardiovascular benefits, reduce body weight, and systolic/diastolic blood pressure in T2DM patients. The glucose-lowering effect provided by SGLT2 inhibitors decreases ROS production (both cytosolic and mitochondrial) and protects the integrity of the mitochondrial function through decreasing AGEs generation, inhibiting NOX activity, lowering HbA1c levels, stimulating antioxidative systems, elevating antioxidative enzyme levels, and decreasing inflammation [26,27,28,29]. Improved mitochondrial functions by SGLT2 administration were demonstrated in the reduced mitochondrial ROS production, restored mitochondrial membrane potential, increased ATP generation, equilibrium of mitochondrial morphology related proteins, and decreased cell death [30]. Here, in this review, we will discuss the involvement of mitochondrial related oxidative stress in the development of PD, the potential shared cellular mechanisms between T2DM and PD, and possible benefits provided by antidiabetic agents for disease modification of PD especially focusing on SGLT2 inhibitors.

## 2. Mitochondria Biology and Oxidative Stress and Mitochondrial Dependent Cell Death

Mitochondria are essential for life. Centered as the main cellular ATP generator through the OXPHOS mechanism and positioned at the heart of cellular metabolism this organelle is complex, dynamic, and communicative within the cellular society through various signaling pathways. Paradoxically the mitochondria are also essential for cell death, and when there is excessive mitochondrial regulation of cell death in neurons, neurodegeneration may occur.

### 2.1. Mitochondrial Biology

The mitochondria are made up of two membranes encompassing two compartments: the mitochondrial matrix, the mitochondrial inner membrane (MIM), the intermembrane space (IMS), and the mitochondrial outer matrix (MOM). The MIM has many invaginations termed cristae which harbor the ETC complexes (complex I to IV) and the F_1_F_0_-ATP synthase. The matrix is the site of multiple energy metabolism pathways whose metabolites (nicotinamide adenine dinucleotide [NADH] and flavin adenine dinucleotide [FADH_2_]) are oxidized to generate electrons which are fed to the ETC complexes. These electrons are then passed through the complexes until transferred from complex IV to the terminal acceptor, oxygen, which is reduced to water. As electrons are transferred from the donor to the more electronegative acceptor, energy is released to enable the ETC complexes to pump protons from the matrix across the MIM to the IMS. As MIM is impermeable to protons, an electrochemical gradient is established across the MIM called the mitochondrial membrane potential (ΔΨ_m_). The resulting proton gradient drives proton flow from the IMS through the F_1_F_0_-ATP synthase back to the matrix and adenosine diphosphate (ADP) is phosphorylated to ATP. This coupling of oxidation electron transfer through the ETC and the final phosphorylation of ADP is known as the OXPHOS [31]. Within each mitochondrial matrix are 2–10 copies of genome independent from that of the nucleus, the mitochondrial DNA (mtDNA). Each set of the genome encodes 37 genes for 13 polypeptides that are key components of the OXPHOS system, a 16S rRNA (large ribosomal unit), a 12S rRNA (small ribosomal unit), and 22 mitochondrial transfer RNAs [32]. 

### 2.2. Mitochondrial ROS

Although mitochondria generate most of the cellular ATP effectively, they are the greatest consumer of cellular oxygen, which make them the main generators of ROS as byproducts. Under physiological conditions, 0.2–2% of the electrons in the ETC leak out during electron transmission [16], especially via the mitochondrial complexes I and III [16]. Leaked electrons are taken up by O_2_ to form the primary ROS, superoxide anion radical O_2_^•−^ which are rapidly dismutated to the more stable hydrogen peroxide (H_2_O_2_) by manganese (Mn)-superoxide dismutase (Mn-SOD/SOD2) in the mitochondria. H_2_O_2_ can then be further detoxified to harmless water with the help of antioxidative enzymes such as glutathione peroxidase (GPx). However, in the presence of transition metal cations such as ferrous ion (Fe^2+^) or Cuprous ion (Cu^+^), H_2_O_2_ can react with O_2_^•−^ to form the harmful hydroxy radical (HO^•^) via the Fenton reaction [33,34]. Murphy et al. have well-reviewed the topic on mitochondrial ROS production and showed that apart from the OXPHOS, ROS are also produced in other sites of the mitochondria (matrix, IMS, and MOM) [35]. Equilibrium must be kept between ROS production and cellular antioxidative systems (antioxidative enzymes and antioxidants) to prevent oxidative damage. Among these, the main antioxidative defense enzymes for superoxides are the superoxide dismutases (SODs), which dismutate O_2_^•−^ into H_2_O_2_ and O_2_ with the help of cofactors such as CuZn and Mn [36]. Three isoforms of SODs exist—copper/zinc (Cu/Zn)-SOD (SOD1) is in the cytoplasm and IMS, Mn-SOD (SOD2) is located in the mitochondrial matrix, and Cu/Zn-SOD (SOD3) is extracellularly located [37]. Mitochondrial H_2_O_2_ is eliminated by the scavenging of the NADPH-dependent glutathione (GSH) and thioredoxin-2 (Trx2) antioxidative systems. Another H_2_O_2_ scavenger, catalase, mainly present in the cytoplasm, is found at very low amounts in brain and heart mitochondria, but more abundant in liver mitochondria [38]. Disequilibrium between the generation of ROS and antioxidative systems causes oxidative stress which brings about damage to macromolecules such as proteins, lipids, and DNA. This may subsequently lead to cellular dysfunction or even cell death [39].

### 2.3. Mitochondria Dynamics, Autophagy, and Mitochondria Mediated Intrinsic Apoptosis

Mitochondria are dynamic organelles and their functions rely on the constant morphological alterations, motility, and positioning that support mitochondrial stability, abundance, distribution, and quality for cells to cope with challenging environments [40]. These organelles form a complex interconnected network and display a diverse range of morphological structures. Through small-scale fusion, they can share and complement membrane, matrix protein, and possible mtDNA [41]. Other effects of mitochondrial fusion include increased energy production, increased cellular proliferation, and protection against apoptotic stress [42]. The three main proteins involved in the fusion of mammalian mitochondria are the GTPases optic atrophy 1 (OPA1) and the two mitofusins (MFN1 and MFN2) [43]. On the contrary, the mitochondria are able to segment dysfunctional portions through mitochondrial fission and enable the degradation of damaged daughter mitochondria via autophagy, also termed mitophagy [44]. The central protein in mitochondrial division is another GTPase, the dynamin-related protein 1 (Drp1). Mitochondria being critical for cell survival, it is essential for the organelle to maintain a healthy network through mitochondrial quality control [45]. This is dependent upon a healthy lysosomal system for mitophagy and mitochondrial-derived vesicles (the degradation of damaged mitochondrial proteins via vesicle trafficking to the lysosome) [45]. In addition to morphological changes and quality control, mitochondria are also dynamic in trafficking through the cell via interaction with the MOM-localized GTPase-like proteins MIRO1/2 along microtubules for distribution [46]. Another function of the mitochondria is to assist in spatio-temporal organization of cellular calcium (Ca^2+^) homeostasis which signal many intracellular processes in health and disease [47]. Ca^2+^ uptake into the mitochondria is driven by ΔΨ_m_ generated via the ETC and Ca^2+^ [47] enters the MOM through the voltage-dependent anion channel (VDAC) and the MIM mainly through the mitochondrial Ca^2+^ uniporter (MCU). Ca^2+^ exit is rapid and occurs via the Na^+^-Ca^2+^ and the H^+^-Ca^2+^ exchangers (NCLX and HCX) [48]. When the mitochondria are under stress, initial defect can be compensated for by mitochondrial dynamics via increased quality control, as well as additional signals sent to other cellular organelles for survival such as through the mitochondrial uncoupled protein response [49,50]. However, when stress is overwhelming, the mitochondria can also send signals to induce mitochondrial-dependent intrinsic apoptosis. Mitochondrial outer membrane permeabilization (MOMP) is the ultimate step in the various apoptotic signal transduction pathways which converge on the mitochondria [51]. Mechanisms underlying these mitochondrial-dependent apoptosis has also been implicated in the pathogenesis of neurodegenerative diseases such as PD as well as T2DM [48,52,53]. Studies focused on targeting mitochondria in PD propose mitochondrial permeability transition pore (mPTP) inhibition in numerous PD adjuvant drugs such as pramipexole, safinamide, ropinirole, rasagiline, and minocycline [52]. One of the representative systems proposed to be responsible for the MOMP is the mPTP [51]. The mPTP is a non-specific channel located in the MIM which can exhibit transient, moderate, or long-lasting openings [54,55]. In the case of mitochondrial ROS and/or Ca^2+^ overload, long-lasting opening of mPTP is triggered. This allows a rapid and massive passage of ions and large molecules, which can dissipate ΔΨ_m and_ result in cell death [53]. The precise molecular composition and identity of the mPTP is highly controversial but candidates include the adenine nucleotide translocase (ANT), the voltage dependent anion channel (VDAC), spastic paraplegia 7 (SPG7), phosphate carrier (PiC) and components of the ATP synthase [54]. Another signaling pathway of mitochondrial-dependent intrinsic apoptosis is through triggering of proapoptotic BH3-only proteins with Bcl-2-associated X protein (BAX) and B-cell lymphoma 2 homologous antagonist killer (BAK) to cause MOMP, the release of proapoptotic factor cytochrome c from the IMS, activation of apoptotic peptidase activating factor 1 (Apaf-1), subsequent activation of caspase cascades (initiator caspase 9, and terminal executioner caspases 3 and 7) [55,56], and eventually cell death [57]. 

## 3. The Linkage between DM and PD

DM is characterized by defective glucose metabolism and is the most widespread metabolic disorder. The disease affects over 400 million adults worldwide with a doubling prevalence since 1980 related in part to the rise of overweight and obese in the population [58]. As early as 60 years ago, Schwab et al. observed a connection between PD and DM and suggested the presence of DM may contribute to the rapidity of progression and reduce the chances of a favorable prognosis in PD [59]. Later on, a prospective study based on the Finnish population associated for the first time T2DM with an increased risk of PD (odds ratio = 1.85 [95% CI 1.23–2.80]), in 2007 [60]. Thereafter, investigations in Europe, the US, and Asia substantiate these findings and recent cohorts evidence that pre-existing DM is a risk for younger-onset PD and affect both motor progression and cognitive decline [61,62,63]. In idiopathic PD subjects, the presence of DM results in a greater rate of cognitive decline, associated with higher white matter atrophy during a 3-year period of follow-up MRI imaging and neuropsychological assessments [64]. Impaired glucose tolerance and hyperglycemia were more commonly present in non-diabetic parkinsonian patients compared with the age-matched control group [65]. The brain is an insulin-sensitive organ with insulin receptors identified in several regions of the brain [66]. These insulin signal pathways regulate important physiological effects including neuronal development, glucoregulation, body weight, and cognitive processes [67]. Additionally, insulin mRNA was found in the periventricular nucleus of the rat hypothalamus [68] and the finding that there is a higher concentration of C-peptide in the postmortem brain than in the blood of human cadavers further suggests that insulin is produced locally in the CNS [69,70]. As insulin in the brain contributes to the control of nutrient homeostasis, cognition, memory, as well as neuromodulatory and neuroprotective effects, alterations of these functional activities may contribute to the manifestation of clinical entities, including central insulin resistance and T2DM [67]. Central insulin resistance favors an adaptive increase in food intake with resulting peripheral alteration of glucose homeostasis [71,72]. Resulting elevation of free fatty acids signals the release of pro-inflammatory cytokines, which then activate insulin inhibition signaling and the further promotion of insulin resistance [73]. Several studies have also suggested that brain mitochondrial dysfunction may be one of the underlying mechanisms contributing to brain insulin resistance and cognitive impairment in the obese condition [74]. In conjunction, insulin receptors are found in the basal ganglia and substantia nigra and growing evidence suggests insulin plays an essential role in regulating dopaminergic transmission and maintenance of synapse [75]. Additionally, patients with PD show marked loss of insulin receptor mRNA in the substantia nigra with increased insulin resistance compared with age-matched control [76]. A critical component of intact insulin signaling is the phosphorylation of insulin receptor substrate-1 (IRS-1) on serine residues which prevent insulin/IGF-1 binding to the insulin receptors and subsequent activation of downstream effectors. Central insulin resistance in AD has proposed an association with elevated levels of phosphorylated IRS-1 at serine residues 636 and 616 [77]. The finding that levels of IRS-1 pSer312 are elevated in blood of PD mice models and blood neuron-derived extracellular vesicles of PD patients may indicate mechanisms of insulin resistance in PD [78,79]. Taken together, therefore, the key DM feature, systemic insulin resistance is also present in the brain of PD patients [80,81].

Indeed, in a non-diabetic PD cohort by Hogg et al. almost two-thirds of the individuals were found to be insulin-resistant (HOMA-IR ≥ 2.0 and/or HbA1c ≥ 5.7), though often with normal fasting glucose and glycated hemoglobin (HbA1c) [82]. Most recently, Markaki et al. report HbA1c levels outside the window of euglycemia (i.e., <31 and >41 mmol/mol) were associated with faster motor symptom progression, independent of age and vascular risk factors, and euglycemia indicates more favorable motor outcome in PD [83]. The cross-talk between glycometabolic derangement and inflammatory response may be important participants in PD motor and cognitive decline. In addition to aging, an increase in HbA1c levels had been shown to be associated with central dopaminergic activity decline in non-diabetic persons [84,85,86].

Linkage between β-cell dysfunction and neurodegeneration during the aging process has been shown by studies with the purpose to clarify the interrelationship between glycometabolic derangement, mitochondrial dysfunction related oxidative stress, inflammatory response, and PD via investigating dietary effect on PD risk [87,88,89]. In agreement with this, the ketogenic diet containing very low carbohydrates was reported to be anti-inflammatory and neuroprotective against mitochondrial complex I inhibitors in both MPTP and 6-OHDA neurotoxic PD animal models [90,91]. Another study also suggested a dietary pattern consisting of high intakes of vegetables, fruits, and fish is associated with a decreased risk of PD in Japanese by measuring overall cumulative dietary pattern [92]. However, an inverse relationship between dietary glycemic index and PD was reported from another study in Japan which did not support a higher carbohydrate diet is related to PD [93].

### 3.1. DM and PD Shared Common Pathogenesis- Mitochondrial Degeneration and Oxidative Damage 

Like other degenerative diseases, mitochondrial dysfunction and oxidative damages also play a crucial role in the development of DM. The ROS generated from mitochondria are the most important origin of oxidative stress in DM, although there are multiple sources of oxidative stress in DM involving non-enzymatic (mostly glucose auto-oxidation and formation of advanced glycation end products (AGEs)), enzymatic (including the polyol pathway and the hexosamine biosynthetic pathway), and mitochondria [94]. Long-term high blood sugar increases the ROS generating enzymes NADPH oxidases (NOX), some of which are subcellular located on the mitochondria [95]. The resultant mitochondrial-induced oxidative stress causes further inhibition of mitochondrial respiratory chain enzymes, leading to initiation of lipid peroxidation, inactivation of glyceraldehyde-3-phosphate dehydrogenase, and oxidative modifications of proteins [96]. Normalizing levels of mitochondrial ROS decreases cellular oxidative damage and prevents glucose-induced activation of protein kinase C, formation of advanced glycation end-products, sorbitol accumulation, and NF-kappa B (NF-κB) activation in cultured bovine aortic endothelial cells [97]. In addition, DM-related increases in free fatty acid production and influx can lead to elevation of ROS generation which further damages the mitochondria and causes deterioration of β-cell function [98,99]. It is suggested that perturbed insulin metabolism may both impair mitochondrial function and elevate ROS production, thus promoting increased oxidative stress and initiating a vicious cycle of metabolic defects. Interestingly, apart from its antidiabetic functions, metformin has antioxidative properties due to its weak mitochondrial complex I inhibiting capability [100,101]. All these findings support that mitochondria integrity is crucial in DM.

From a genetic point of view, variations of mtDNA can potentially alter mitochondrial function and thus be possible genetic etiology for DM and PD [89,102]. The human mitochondrial genome is very compact and more than 90% of human mtDNA are composed of coding sequences without containing introns but with a single major non-coding region, called the D-loop region [103]. Due to the proximity of the mtDNA to ROS produced in situ by the mitochondria and lack of protective histones, human mtDNA is more prone to mutations than the nuclear DNA [104]. These polymorphisms are mostly found within the coding region of the mitochondrial genome [105]. For example, the pathogenic mtDNA A3243G mutation in the tRNA^Leu (UUR)^ (MTTL1) gene is harbored by approximately 80% of cases of mitochondrial encephalomyopathy, lactic acidosis and stroke-like episodes syndrome (MELAS), and also maternal inherited diabetes mellitus [106,107,108,109,110,111]. The non-pathogenic mtDNA T16189C variant, a polymorphism located in the mitochondrial D-loop region that regulate mtDNA replication is associated with increased oxidative damage, altered antioxidative status in T2DM patients, metabolic syndrome, higher fasting insulin concentration, insulin resistance index, and lacunar cerebral infarction in the Asian population [112,113,114,115,116]. mtDNA haplogroups represent the major branch points to map the evolutionary path of the human female lineage, based on the mitochondrial phylogenetic tree [117]. Our group has reported that the haplotype B4 (carrying the T16189C variant) is associated with T2DM (odds ratio [OR], 1.54 [95% CI 1.18–2.02]; *p* < 0.001) in the Chinese population, whereas subjects harboring haplogroup D4 have borderline resistance against DM generation (0.68 [0.49–0.94]; *p* = 0.02) [112]. Additionally, we recently reported an association between mitochondrial haplogroup F1 and risk of ischemic stroke (OR 1.72:1.27–2.34, *p* = 0.001) in a patient study enrolling 830 Taiwanese ischemic stroke patients and 966 normal controls [118]. In the same study, cytoplasmic hybrid (cybrid) cells, fusion of mtDNA-depleted ρ^0^ cells with platelets from donors harboring 20 of the major haplogroups in the Asian population, were cultured. Tsai et al. demonstrated that mitochondrial haplogroup F1 cybrids were associated with decreased oxygen consumption, higher mitochondrial ROS production, and lower mitochondrial membrane potential [118]. All these data support the finding that mitochondrial dysfunction caused by mitochondrial genetic variation is related to the risk of T2DM and the sequential elevated oxidative stress may lead to further neurological conditions. Similar to T2DM, alteration of mitochondrial function caused by mtDNA variations have also been reported to be associated with PD risks. Our group has reported that subjects harboring the mitochondrial haplogroup B5 have resistance against PD (odds ratio 0.50, 0.32–0.78; *p* = 0.002). Furthermore, a composite mtDNA variant group consisting of A10398G and G8584A at the coding region was also found to have resistance against PD [119]. Alike results were also reported later by another group from a Chinese population that haplogroup B5 carriers were more resistant to PD and subjects carrying haplogroup A5 were more susceptible to the disease [120]. Accordingly, mitochondrial dysfunction related to mitochondrial genetic variation contributes to the development of degenerative diseases including both T2DM and PD.

Thus, mitochondrial dysfunction and oxidative damage are shared by both neuron and islet cell degeneration, and the usage of antioxidants or antidiabetic agents may potentially provide protection and could halt the speed of declination [121].

### 3.2. The Usage of Anti-Diabetes Agents in Slowing PD Progression

As stated above, a tight linkage between PD and DM has been reported [63,122]. Repurposing existing antidiabetic drugs is emerging as potential candidates for the development of promising therapeutic approaches for PD [123]. For example, traditional glucose-lowering medication metformin was demonstrated to be associated with a lower incidence of PD [124]. Neuroprotective effects of metformin on PD have also been demonstrated in a large amount of animal and cellular studies through mechanisms including inhibiting α-synuclein aggregation, reversing mitochondrial dysfunction, attenuating oxidative stress, modulating autophagy, and activation of the AMP-activated protein kinase (AMPK) pathway [125,126,127]. Neuroprotective effects of glitazones, a class of peroxisome proliferation-activated receptor gamma (PPARɣ) agonist, have been shown in cellular and animal studies. Rosiglitazone, a glitazone, arrested severe decline of striatal dopamine and partial degeneration of the SNc through reverting PPAR-γ overexpression in microglia, countering the increase in CD11b immunoreactivity, and restoring tumor necrosis factor α (TNF-α) expression down to control levels in a progressive MPTP/probenecid chronic mouse model of PD [128]. Pioglitazone, another glitazone, was also shown to reduce the expression of matrix metalloproteinases (MMP 3 and 9), which are known microglial activators as well as a direct anti-inflammatory on microglia, in the substantia nigra of 6-hydroxydopamine (6-OHDA) rodent model of PD [129]. The use of glitazones has also been shown to be associated with a 28% lower rate of PD compared to those prescribed other antidiabetic agents in individuals with DM in a large population-based cohort study from the UK [130]. Despite a definite conclusion needing further confirmation, a recent exploratory meta-analysis supports the value of using glitazones in reducing PD incidence in diabetic patients [131].

Another antidiabetic drug, the incretin hormone glucagon-like peptide-1 receptor agonists (GLP-1RA), acts as an incretin mimetic and increases insulin secretion from the pancreatic beta cells. Accumulating evidence suggests that the GLP-1 RAs can cross the blood-brain barrier to influence several neuronal pathways, including inhibitory effects on inflammation, promotion of mitochondrial biogenesis, neurotrophic effects, stimulation of neurogenesis, and restoration of neuronal insulin signaling [132,133,134]. In different preclinical models of PD, GLP-1 RAs showed neuroprotective effects, influencing motor activity, dopaminergic neurons, cortical activity, and energy utilization in the brain [135]. Our group has also demonstrated the neuroprotective effects of liraglutide, in improving mice neurobehavior and increasing neuronal survival in the substantia nigra through affecting the mitochondrial morphology, enhancing autophagy flux, decreasing α-synuclein aggregation, and decreasing oxidative stress in an acute MPTP mice PD model [136]. In an open-label trial (NCT01174810), injections of exenatide improved overnight off-medication motor symptoms and dementia in 44 PD patients.

The other oral hypoglycemics, DPP-4 inhibitors (or gliptins) can enhance the bioavailability of GLP-1 for the purpose of increasing incretin levels. For example, the DPP4 inhibitor sitagliptin could ameliorate rotenone-induced decrease of dopamine production and improve motor function in a rotenone PD rat model, as discussed above [137]. Supporting this, treatment with DPP4 inhibitors confer beneficial effects on the baseline nigrostriatal dopamine degeneration and long-term motor outcomes in diabetic patients with PD and may extend its role into non-diabetic patients with PD [138]. Similar results have also been reported by Brauer et al. that the use of DPP4 inhibitors and/or GLP-1 RA is associated with a lower rate of PD compared to the use of other oral antidiabetic drugs [139].

## 4. SGLT2 Inhibitors and PD

### 4.1. SGLT2 Inhibitors

**Figure 1:**
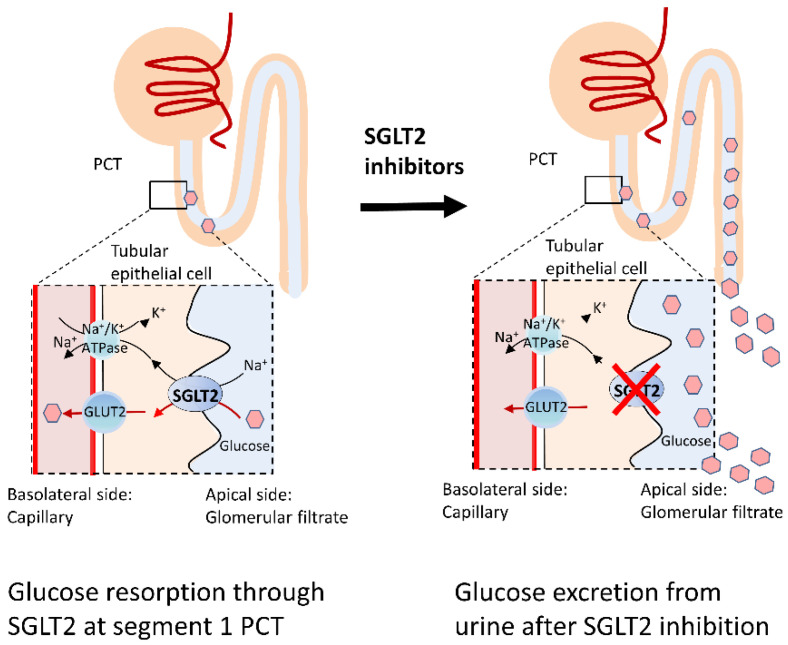
Resorption of filtered glucose in the proximal convoluted renal tubules. Filtered glucose in the kidneys is reabsorbed via coordinated functions of Na^+^/K^+^ ATPase and SGLT. Up to 90% of glucose resorption is through the SGLT2 located in the first segment of the proximal convoluted tubule. Addition of the SGLT2 inhibitors results in increased urinary glucose excretion and therefore reduced serum glucose levels. (Abbreviations: SGLT, sodium-glucose cotransporters; GLUT2, Glucose transporter 2; PCT, proximal convoluted tubule).

SGLT2 inhibitors, also called gliflozins, are a new class of medications that inhibits the reabsorption of glucose in the kidney and therefore lower blood sugar by inhibiting SGLT2. In 2013, canagliflozin became the first U.S. Food and Drug Administration (FDA) approved gliflozin. The secondary-active glucose transporter, SGLT2, are predominately located on the brush border of epithelial cells in segments 1 and 2 of the proximal convoluted tubule of the kidney and are responsible for more than 90% of the reabsorption of the glucose filtered at the glomerulus. Glucose molecules are absorbed across the SGLT2 against the concentration gradient across epithelial cells of the kidney using energy provided by the sodium gradient across the brush-border membrane, which is maintained by the Na^+^/K^+^ ATPase [140]. By selectively blocking these glucose transporters, SGLT2 inhibitors effectively block glucose reabsorption leading to enhanced glycosuria and improved diabetes control independent of insulin [141] (Figure 1). In addition to improving glycemic control, these drugs also reduce body weight and blood pressure related to early natriuresis [142]. The benefits of SGLT2 inhibitors in cardiovascular and renal outcomes have been reported in large outcome studies such as the EMPA-REG OUTCOME trial [143], CANVAS (CANagliflozin cardiovascular Assessment Study) [144], DECLARE–TIMI 58 [145], Comparative Effectiveness of Cardiovascular Outcomes (CVD REAL) real-world study [146], and DAPA-HF trial [147]). These beneficial effects seem to extend beyond improved glycemic control and underlying mechanisms are still being extensively investigated. Proposed beneficial mechanisms of SGLT2 inhibitors include a reduction in proinflammatory cytokines, a shift towards FFA and ketone bodies as the metabolic substrate, reduced oxidative stress, reduced glomerular hyperfiltration, suppression of advanced glycation end-product signaling, and increased hepatic glycogen depletion with restoration of adequate catabolic periods [140,148]. Recent studies have also demonstrated the involvement of SGLT2 inhibitor in restoring mitochondrial function and activating the autophagy-lysosomal pathways [149], deficits in both these pathways have been suggested to be pivotal in the development of neurodegenerative diseases such as PD. This glucose transporter, SGLT2, has also been found in the hippocampus, cerebellum, and at blood-brain barrier endothelial cells [150]. The particular anatomical distribution, along with the pathophysiology similarities shared between PD and DM, paved the strategy of using SGLT2 inhibitors for PD disease-modifying purposes.

### 4.2. Protective Mechanisms of SGLT2 Inhibitors on PD and DM: Antioxidative Activities and Mitochondria Protection 

**Figure 2:**
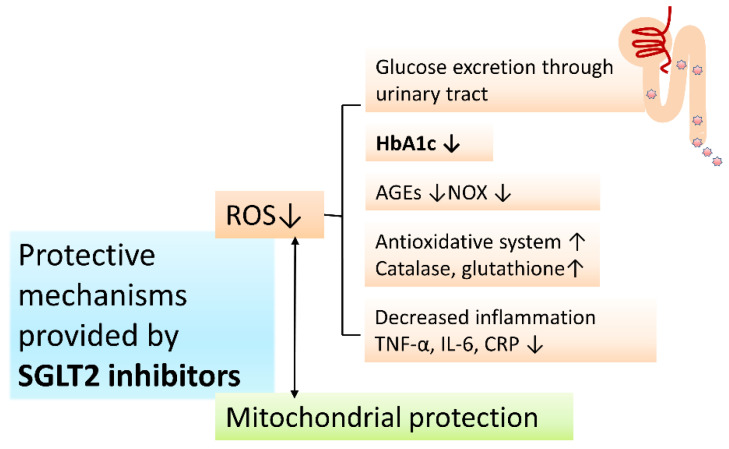
SGLT2 inhibitors decrease ROS levels and maintain the integrity of the mitochondrial network. Protective mechanisms provided by SGLT2 inhibitors is mainly through decreasing ROS levels and protecting the integrity of the mitochondrial network. ROS level maintenance is kept through the urinary excretion of glucose decreasing downhill stimulation of ROS production through hyperglycemic related mechanisms, decreasing AGEs generation, inhibiting NOX acitivity, lowering HbA1c levels, stimulating antioxidative systems, elevating antioxidative enzyme levels, and decreasing inflammation. (Abbreviations: AGEs, Advanced Glycation End Products; CRP, C-reactive protein; HbA1c, glycated hemoglobin; NOX, NADPH oxidases; ROS, reactive oxygen species; SGLT2, sodium-glucose cotransporter 2; TNF-α, tumor necrosis factor-α; IL-6, interleukin-6).

As has been stated above, there is an increased risk of PD in DM patients and the motor/non-motor symptoms are worse among PD patients with DM [151]. Through excretion of filtered glucose at the proximal convoluted tubules, SGLT2 inhibitors attenuate hyperglycemia-induced neuronal ETC overload and ROS production. With upsurge of intracerebral glucose in hyperglycemic status, more fuel is given to the tricarboxylic acid cycle (TCA) through glycolytic pathways in neurons, resulting in increased production of electron donors, and amplified flux of electron donors toward OXPHOS [123]. Additionally, surrounding astrocytes generate lactate from glycolysis which enter neurons, are converted to pyruvate, and power the TCA cycle further [152]. The end result is ETC overload and eventual increase in the proton gradient across the MIM which, in turn, impairs the normal transportation of electrons through the mitochondrial complexes causing increased leakage of electrons and the formation of ROS [35]. Of note, under hyperglycemic conditions, the NADH/NAD^+^ ratio is high in the matrix, which favors the formation of O_2_^•−^ inside the matrix [152]. Thus, oxidative stress related mitochondrial dysfunction can be partially attenuated by effective antiglycemic effect provided by SGLT2 inhibitors in the CNS [148]. In addition, administration of SGLT2 inhibitors could elevate plasma levels of ketone bodies which promotes oxidation of the mitochondrial coenzyme Q as an efficient energy source and could reduce the intracellular sodium overload to improve mitochondrial energetics and oxidative defense [153] (Figure 2).

**Figure 3:**
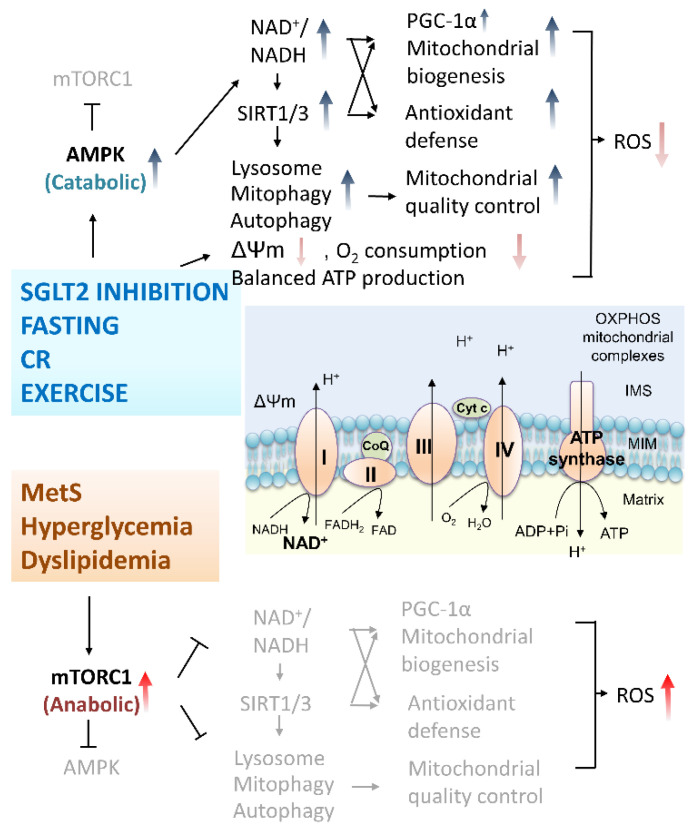
Adjustments of mitochondrial bioenergetics and quality control in subjects under different energy status. Healthy individuals maintain regular metabolic adjustments between anabolic (fed) and catabolic (fasted) cycling. In the case of SGLT2 inhibition, fasting, CR, and long-term exercise, the catabolic mediator AMPK is stimulated while mTORC1 is inhibited. This increases in NAD+/NADH ratio elevates SIRT1 and SIRT3 resulting in downstream elevated PGC-1α expression, increased mitochondrial biogenesis, enhanced cellular antioxidative mechanisms, mitophagy activation, decreased O_2_ demand, lowering but not dissipating ΔΨm, without disturbing OXPHOS efficiency and ATP production. Together, mitochondrial quality control is enhanced and ROS levels are reduced. In metabolic syndrome patients, hyperglycemia or/and dyslipidemia activate the major anabolic regulator mTORC1 while AMPK is inhibited. This results in increased ROS production, reduced mitochondrial quality control leading to further increased cellular oxidative stress. (Abbreviations: AMPK, 5’-adenosine monophosphate (AMP)-activated protein kinase; ATP, adenosine triphosphate; CoQ, Coenzyme Q; Cyt c, cytochrome c; CR, calorie restriction; IMS, intermembrane space; MIM, mitochondrial inner membrane; ΔΨm, mitochondrial membrane potential; mTORC1, mammalian target of rapamycin complex I; NAD+/NADH, ratio of oxidized and reduced forms of nicotinamide adenine dinucleotide; OXPHOS, oxidative phosphorylation; PGC-1α, peroxisome proliferator-activated receptor-gamma coactivator (PGC)-1alpha; SGLT2, sodium-glucose cotransporters 2; SIRT1, Sirtuin-1).

Normalization of hyperglycemia provides further mitochondrial protection via the induction of mitochondrial quality control through autophagy/mitophagy and lysosomal degradation. Lean healthy adults possess regular metabolic adjustments between anabolic (fed) and catabolic (fasted) cycling. In the nutrient-rich anabolic state, glucose is the primary fuel, producing energy through glycolysis and the mitochondrial OXPHOS system [149]. Nutrient-poor catabolic periods mostly occur in overnight fasting with decreased circulating glucose and insulin while elevated glucagon maintains physiological glucose levels in the brain. In this catabolic period, general fuel outside of the brain is switched from glucose to free fatty acid oxidation and low insulin leads to increased lipolysis supplying free fatty acid for β-oxidation. Low levels of insulin and circulating amino acids induced by fasting inhibit the central regulator of cell metabolism the mammalian target of rapamycin complex 1 (mTORC1) which enhances the autophagy/mitophagy and lysosomal degradation to supply amino acids for gluconeogenesis. Different from healthy people, T2DM patients have characteristic lower insulin, paradoxical glucagon, high plasma glucose, and high free fatty acid. Metabolically, these patients are unable to switch from the mainly glucose oxidation state (fed) to the mainly fatty acid oxidation state (fasted) [154]. The inhibition of SGLT2 has been shown to partially restore this metabolic flexibility due to the glucose disposal through the renal system, depletion of glycogen in the liver, resulting in gluconeogenesis utilizing circulating amino acid, and inhibiting mTORC1 [155]. As mitophagy is inhibited during fed state (activate mTORC1 for anabolic processes including mitochondrial biogenesis) and enhanced during fasting (inhibit mTORC1 for catabolic autophagy/lysosomal processes including mitophagy), induction of mitophagy potentially provides neuroprotection through augmenting the clearance of damaged mitochondria in the central nervous system [156,157]. Nicely reviewed by Esterline et al., the authors proposed that SGLT2 inhibitors induce periods of overnight gluconeogenesis which provides the catabolic state critical for quality control and renewal of damaged cellular organelles [149] (Figure 3).

### 4.3. Protective Evidence of SGLT2 Inhibitors for Complications of Metabolic Syndromes

**Figure 4:**
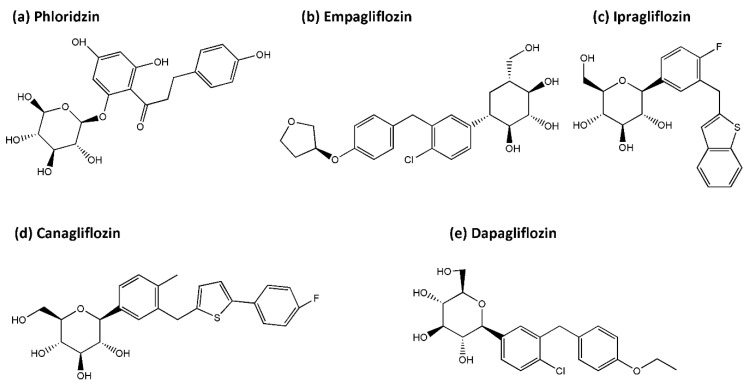
Chemical structure of the SGLT2 inhibitors discussed within this review: (**a**) Phloridzin (**b**) Empagliflozin (**c**) Ipragliflozin (**d**) Canagliflozin (**e**) Dapagliflozin (Abbreviations: SGLT2, sodium-glucose cotransporter 2).

The SGLT2 inhibitors approved by the FDA, European Medicines Agency (EMA), Japan Pharmaceuticals and Medical Devices Agency (PMDA), and similar bureau in other countries include empagliflozin, dapagliflozin, canagliflozin, ertugliflozin, ipragliflozin, tofogliflozin, luseogliflozin, and remogliflozin [158]. The general structure of this class of drugs is relatively similar and falls into the C-glucoside classification of the SGLT2 inhibitors with a glucose sugar and an aromatic group in the β-position at the anomeric carbon [159,160]. Below, we will summarize the most studied SGLT2 inhibitors, including the first found naturally occurring phloridzin as well as the pharmaceutical empagliflozin, ipragliflozin, canagliflozin, and dapagliflozin (Figure 4).

The first recognized SGLT2 inhibitor, phloridzin (a nonselective inhibitor of both SGLT1 and SGLT2) was introduced in the 1950s [161]. This polyphenol is easily assessable in apples, has antioxidative and anti-neuroinflammatory effects, and has been suggested to be a potential candidate for mitochondrial-directed flavonoid therapy in neurodegenerative diseases [162,163]. Phlorizin has been demonstrated to protect against endothelial dysfunction in T2DM by activating the phosphatidylinositol 3′-kinase (PI3K), Akt, and endothelial nitric oxide synthase (eNOS) (PI3K/Akt/eNOS) signaling pathway [9]. Studies also showed that phloridzin was able to restore the activities of antioxidative enzymes such as catalase and glutathione peroxidase (GPx) in kidneys of diabetic rats [164].

The SGLT2 inhibitor empagliflozin is known for its cardiovascular- and reno-protective effects [143]. This type of medication is demonstrated to reduce high glucose-induced renal cell apoptosis and improve mitochondrial functions by reducing mitochondrial ROS production, restoring mitochondrial membrane potential, increasing ATP generation, and modulating mitochondrial morphology related proteins [30]. In murine myocardium and cultured cardiomyocytes, Maejima demonstrated empagliflozin to play a protective role by promoting mitophagy through binding with non-SGLT2 protein(s) localizing in mitochondria [153]. Chronic empagliflozin administration effectively decreased renal oxidative stress, tissue inflammation, and improved insulin sensitivity in diabetic rats via mechanisms including decreased methylglyoxal levels in diabetic neuropathy, reduced AGE/RAGE signaling, improved ^•^NO/cGMP signaling, upregulated redox regulated enzyme mitochondrial aldehyde dehydrogenase (ALDH-2), and decreased epigenetic activation of Nitric Oxide Synthase (NOS2) and IFNγ [28,165]. In diabetic rats, empagliflozin reduced the mortality rate of post-acute myocardial infarction (MI) animals with modification of cardiac metabolomes through SIRT3 upregulation which restores glucose oxidation, increases ketone oxidation, and decreases fatty acid oxidation, enabling the maintenance of the ATP level in the myocardium. In addition, antioxidative enzymes including SOD2 and catalase are enhanced [166]. Empagliflozin has also been shown to upregulate levels of sestrin 2 which consequently increases downstream AMPK phosphorylation, inhibits mTOR signaling, and enhances the primary endogenous antioxidant defense nuclear factor erythroid 2–related factor 2 (Nrf2)/ heme oxygenase-1 (HO-1) pathway in cardiomyocytes [167]. Through activation of STAT3 and downregulation of iNOS (a key contributing molecule in nitrosative stress) and IL-6, empagliflozin provides antioxidative and anti-inflammatory effects in a mice and rat cardiomyoblasts model [168]. In T2DM patients, treatment of empagliflozin for 24 weeks enhanced leukocyte antioxidative enzymes expression (glutathione s-reductase and catalase) and elevated serum anti-inflammatory cytokine interleukin 10 (IL-10) levels, with concomitant reduction of inflammatory markers, high sensitive C-reactive protein (hs-CRP) and myeloperoxidase [169].

Ipragliflozin, the first SGLT2 inhibitor approved in Japan, alleviated mitochondrial dysfunction via restoring the levels of mitochondrial fusion proteins Opa1 and Mfn2 and decreased 8-hydroxydeoxyguanosine (8-OHdG), an oxidative DNA damage biomarker, in a high-fat diet rat model [170]. Furthermore, ipragliflozin improved hyperlipidemia, hepatic steatosis, renal glomerular hyperfiltration, albuminuria, and provided hepatic protection with concomitantly reduced liver levels of oxidative stress biomarkers (thiobarbituric acid reactive substances [TBARS], protein carbonyl) and plasma inflammatory markers (IL-6, TNF-α, monocyte chemotactic protein-1 [MCP-1], and CRP) in T1DM rats [171]. 

The SGLT2 inhibitor, canagliflozin, has been shown to possess cardioprotective effects through stimulating antioxidative and anti-inflammatory signaling mediated by inducible nitric oxide synthase (iNOS) and endothelial nitric oxide synthase (eNOS) in cardiomyocytes [172]. Yang et al. demonstrated canagliflozin treatment directly increased kidney and subcutaneous adipocyte cellular energy expenditure by inducing mitochondrial biogenesis, increasing mitochondrial oxidative phosphorylation, fatty acid oxidation, and thermogenesis via AMPK–silent information regulator 1 (Sirt1)–peroxisome proliferator-activated receptor γ coactivator-1α (PGC-1α) signaling pathway in high-fat diet mice [173].

Dapagliflozin, the SGLT2 inhibitor shown to reduce cardiovascular death in DECLARE-TIMI 58 and DAPA-HF clinical trials [145,147], was able to preserve the depolarized mitochondrial membrane potential, alter mitochondrial morphology related protein levels (Mfn-1, Mfn-2, and Fis-1), maintain cytosolic Ca^2+^ homeostasis, and significantly improve cellular oxidative damage, protein-thiol oxidation, and ADP/ATP ratio in cardiomyocytes of insulin-resistant rats with metabolic syndrome [174]. Recently, increasing neuroprotective evidence provided by SGLT2 inhibitors in CNS is emerging. In the following section, study results generated from models of neurodegenerative diseases will be elucidated.

### 4.4. Neuroprotection Provided by SGLT2 Inhibitors

As shown above, data concerning beneficial effects of SGLT2 inhibitors are expanding in many organ systems, neuroprotective effects provided by sglt2 will be summarized below and in Table 1. In the central nervous system, with amelioration of oxidative damage, empagliflozin was also able to attenuate cognitive dysfunction in T2DM mice documented by water maze [175]. Hierro-Bujalance, et al. also demonstrated empagliflozin to reduce vascular damage, limit cortical thinning, reduce neuronal loss, reduce microglial burden in the brain parenchyma, and improve cognitive impairment in a mixed murine model of Alzheimer’s disease and T2DM (db/db mice and APP/PS1xdb/db mice) [176]. The group also noted that empagliflozin could cause limited reduction of senile plaque density, and both soluble and insoluble amyloid β levels in the cortex and hippocampus were reduced [176]. Amin et al. supported this, demonstrating that empagliflozin decreased cerebral infarct volume, suppressed neuroinflammation, oxidative stress, and reduced neuronal apoptosis in brain tissues of hyperglycemic I/R-injured rats [177]. Additionally, Rania et al. showed the ameliorative effect of empagliflozin on neuronal apoptosis via decreasing caspase 3 and HIF-1α/VEGF signaling as well as decreasing infarct volume in a cerebral ischemia/reperfusion (I/R) rat model induced via occlusion and reperfusion of the bilateral common carotid arteries [178]. Arafa et al. suggested canagliflozin prevention of scopolamine hydrobromide-induced memory impairment in rats via cholinergic and monoamines system as observed through the number of arm entry and number of correct alternation in Y maze task and performance in the water maze task [179]. Additionally, dapagliflozin could prevent cognitive decline and preserve synaptic plasticity via attenuating mitochondrial dysfunction, neuronal apoptosis, brain insulin resistance, inflammation, and apoptosis in obese rats induced by high-fat diet. In the same study, Sa-nguanmoo et al. demonstrated treatment with combination of SGLT2 inhibitor (dapagliflozin) and DPP-4 inhibitor (vildagliptin) exerted greater antioxidative effects and improved brain insulin sensitivity compared with single therapy [180]. Apart from the restoration of disturbed DJ-1/Nrf2 pathway, upregulating glial cell line-derived neurotrophic factor (GDNF) and its downstream PI3K/AKT/GSK-3β (Ser9) pathway which counteracts ROS-dependent neuronal apoptosis, dapagliflozin also suppressed neuroinflammation via curbing the activation of NF-κB pathway and TNF-α levels [181]. Erdogan demonstrated dapagliflozin to decrease seizure activity in rats with pentylenetetrazol-induced seizures. This may be connected to the glucose-lowering nature and reduced sodium transport across neuronal membranes provided by the SGLT2 inhibitor which can confer a stabilizing effect against excitability and unwanted depolarization [182]. Tsai et al. performed a meta-analysis to evaluate the effect of SGLT2 inhibitors on risk of stroke and its subtypes. Five prospective RCTs, involving 46,969 participants, on SGLT2 inhibitors with stroke events as the primary endpoint in T2DM patients were subjected to the study (EMPA-REG, CANVAS, DECLARE-TIMI 58, CREDENCE, and VERTIS CV). Results demonstrated that SGLT2 inhibitors have a neutral effect on the risk of stroke and its subtypes (fatal stroke, non-fatal stroke, ischemic stroke, or transient ischemic attack), but a potential protective effect against hemorrhagic stroke was associated with SGLT2 inhibitors a significant 50% reduction compared with placebo (RR = 0.49, 95% CI 0.30–0.82, *p* = 0.007) [183]. Based on the beneficial effects provided by SGLT2 inhibitors, a randomized clinical trial using dapagliflozin on delaying cognition decline in T2DM patients (NCT04304261) is expected to recruit 100 participants since 11 March 2020, and study results are pending.

### 4.5. Potential Usage of SGLT2 Inhibitor in PD Neuroprotective Strategy

With a growing body of research concerning the cellular protective effect provided by SGLT2 inhibition, studies focusing on PD related models are still scarce. The presence of phloridzin could scavenge rotenone evoked superoxide generation, decrease nuclear DNA fragmentation, decrease lipid peroxidation, restore mitochondrial membrane potential, and reduce mitochondrial-dependent apoptosis, in a rotenone induced PD neuronal cell model [184]. Administration of dapagliflozin to a rotenone-induced PD rat model attenuated motor dysfunction, documented by the open-field and rotarod, with concurrent diminished α-synuclein expression, improved dopamine secretion, alleviated oxidative stress, and decreased dopaminergic neuronal loss [181]. As such, the study of SGLT2 inhibitors in the neurological system especially in association with PD is just beginning and further studies focusing on the protective mechanism provided by this class of already widely used medication will be expected to thrive.

**Table 1:** Neuroprotective effect of SGLT2 inhibitors.

SGLT2 Inhibitor/ Dose/Time	Model	Findings	Ref.
Empagliflozin/ 0.03% empagliflozin diet/10 wks	db/db mouse, obesity and T2DM model	Enhanced: BDNF in cerebral tissue, Reduced: cerebral oxidative stress, DNA oxidative damage Function: Slowed progression of cognitive impairment,	[175] Lin et al.
Empagliflozin/ 10 mg/kg/day/ 22 wks	APP/PS1xdb/db mice, Mixed AD and T2DM model	Reduced: brain atrophy, senile plaques, amyloid-β levels, Tau phosphorylation, hemorrhage density, microglia burden Function: Enhanced learning, memory	[176] Hierro-Bujalance et al.
Empagliflozin/ 10 mg/kg/day/ 10 wks	T2DM *db*/*db* and lean control female mice	Protects mice brain from severe T2DM-induced ultrastructural remodeling of the neurovascular unit	[185] Hayden et.al.
Empagliflozin/ 10 mg/kg/ 1 h and 24 h after reperfusion	Wistar rats hyperglycemic model + STZ (55 mg/kg)-induced I/R model	Reduced: cerebral infarct volume, neuroinflammation, oxidative stress, neuronal apoptosis Function: enhanced behavioral/ neurological functions	[177] Amin et al.
Empagliflozin/ 1 and 10 mg/kg/ 1 h and 24 h after reperfusion	Wistar rats, transient bilateral common carotid arteries occlusion induced I/R model	Enhanced: HIF-1α, VEGF Reduced: brain apoptotic cell death(↓caspase-3), infarct volume Function: Reduced motor dysfunction, neurological deficit * large dose with better neuroprotective effect	[186] Abdel-Latif et al.
Canagliflozin/ 10 mg/5mL/ vs. Galantamine/ 3 mg/5 mL/ 1st and 13th day	Wistar rats, Scopolamine hydrobromide (C17H21NO4·HBr) induced memory dysfunction model	↓memory dysfunction (Y maze task) ↑hippocampus M1 mAChR	[179] Arafa et al.
Dapagliflozin/ 1 mg/kg/day/ vs. Vildagliptin/3 mg/kg/day/ 4 wks	Wistar rats, HFD model	Reduced: brain mt ROS production, ΔΨm change, mt swelling, neuroinflammation (↓p-NFκB, p65/ NFκB p65 ratio), neuronal apoptosis (↓Bax, Bcl2) Enhanced: brain mitochondrial function, p-IR and p-Akt/PKB ser473 Function: Both drugs: Enhanced insulin sensitivity, prevented cognitive decline. Only dapagliflozin improved hippocampal synaptic plasticity * the combination of two drugs has better effect than single therapies	[180] Sa-Nguanmoo et al.
Dapagliflozin/ 1 mg/kg/day/ 3 wks	Wistar rats, rotenone-induced PD model	Reduced: brain mt ROS production, α-synuclein expression, neuroinflammation (↓p-NFκB, p65/NFκB p65 ratio, TNF-α), striatal neuronal oxidative stress (↓DJ-1, Nrf2, HO-1, ↓GDNF, PI3K/AKT/GSK-3β), neuronal apoptosis (↓Bax, cleaved caspase 3) Preserved striatal dopaminergic neurons Function: Improved neurodegenerative aberrations/motor dysfunction	[181] Arab et al.
Dapagliflozin 75 and 150 mg/kg	Sprague–Dawley rats with pentylenetetrazol-induced seizures	↓seizure activity (EEG SWP, RSS, TFMJ) * higher dose more effective	[182] Erdogan et al.
**Clinical trial data**			
SGLT2 inhibitors	(a systematic review and meta-analysis that included 5 clinical trials) EMPA-REG, CANVAS, DECLARE-TIMI 58, CREDENCE and VERTIS CV	* potential protective effect against hemorrhagic stroke * neutral effect on the risk of fatal stroke, non-fatal stroke, ischemic stroke or transient ischemic attack)	[183] Tsai et al.
dapagliflozin	T2DM patients	cognition decline (Recruiting)	NCT04304261

Abbreviation: SGLT2, sodium–glucose cotransporter 2; Ref., reference; BDNF, brain-derived neurotrophic factor; AD, Alzheimer’s disease; T2DM, Type 2 diabetes mellitus; STZ, streptozotocin; STZ, streptozotocin; I/R, ischemia/reperfusion; HIF-1α, hypoxia-inducible factor 1 alpha; VEGF, Vascular endothelial growth factor; mAChR, muscarinic acetylcholine receptor; HFD, high-fat diet; EEG, electroencephalography; SWP, spike wave percentage; RSS, Racine’s scales scores; TFMJ, time to first myoclonic jerk. *, additional explanation.

## 5. Perspectives

Accumulating evidence has suggested that the T2DM medication SGLT2 inhibitors provide antioxidative effects and mitochondrial protection in human disease models including hepatic, renal, cardiac, and nervous conditions. Studies focused on the disease modification effects of SGLT2 inhibitors in neurodegenerative diseases including PD are still limited. Although effective symptomatic pharmaceutical managements are available, neuroprotective or disease-modifying agents are still an unmet goal for PD. Owing to the consistent evidence of the benefits of SGLT2 inhibitors on a broad spectrum of end points in large randomized trials, potential neuroprotective effects using this antidiabetic drug is expected. Further experiment data and clinical trials are mandatory in the future for additional evidence to support mitochondrial protective drugs such as SGLT2 inhibitors to be used alone or in combination with drugs of different mechanisms for the purpose of neuroprotection in PD.

## Glossary

AD	Alzheimer’s disease
ADP	Adenosine diphosphate
AGEs	advanced glycation end products
AMPK	5’-adenosine monophosphate (AMP)-activated protein kinase
APAF1	Apoptotic peptidase-activating factor 1
ATP	Adenosine triphosphate
ATP13A2	Atpase type 13 a 2
BAK	Bcl2-antagonist/killer
BAX	Bcl-2-associated x protein
Ca^2+^	Calcium
CoQ	Co-enzyme q
CR	Caloric restriction
CVD	Cardiovascular diseases
DJ-1	Daisuke-junko-1
DPP-4	dipeptidyl peptidase-4
ETC	Electric transport chain
FADH_2_	flavin adenine dinucleotide
FBXO7	F-box only protein 7
GLP-1RA	glucagon-like peptide-1
GPX	glutathione peroxidase
GSH	Glutathione
HO•	hydroxy radical
IMS	Intermembrane space
LRRK2	Leucine rich repeat kinase 2
MELAS	mitochondrial encephalomyopathy, lactic acidosis and stroke-like episodes syndrome
Mfn-1 and 2	Mitofusins 1 and 2
MIM	Mitochondrial inner membrane
ΔΨm	Mitochondrial membrane potential
MOM	Mitochondrial outer membrane
MOMP	Mitochondrial outer membrane permeabilization
MPTP	1-methyl-4-phenyl-1,2,3,6-tetrahydropyridine
mtDNA	Mitochondrial DNA
mTOR/mTORC1	Mammalian target of rapamycin/mammalian target of rapamycin complex I
nDNA	Nucleus DNA
NADH	nicotinamide adenine dinucleotide
NF-κB	Nuclear factor kappa-light-chain-enhancer of activated B cells
NOX	Nicotinamide adenine dinucleotide phosphate oxidase
Nrf2	nuclear factor erythroid 2–related factor 2
OPA1	optic atrophy 1
OXPHOS	Oxidative phosphorylation
PD	Parkinson disease
PGC	Peroxisome proliferator-activated receptors (PPAR) γ coactivator
PINK1	Phosphatase and tensin homologue (PTEN)-induced putative kinase 1
PPAR	Peroxisome proliferator-activated receptor
ROS	Reactive oxygen species
rRNA	Ribosomal rna
SGLT2	sodium–glucose cotransporter 2
SOD	Superoxide dismutase
SIRT-1	Sirtuin-1
TCA	Tricarboxylic acid
TNF-α	tumor necrosis factor α
TRX	Thioredoxin

## References

[1] Poewe W., Seppi K., Tanner C.M., Halliday G.M., Brundin P., Volkmann J., Schrag A.-E., Lang A.E. (2017). Parkinson disease. Nat. Rev. Dis. Primers.

[2] Schapira A.H.V., Chaudhuri K.R., Jenner P. (2017). Non-motor features of Parkinson disease. Nat. Rev. Neurosci..

[3] Jankovic J. (2008). Parkinson’s disease: Clinical features and diagnosis. J. Neurol. Neurosurg. Psychiatry.

[4] Abeliovich A., Gitler A.D. (2016). Defects in trafficking bridge Parkinson’s disease pathology and genetics. Nature.

[5] Gorell J.M., Johnson C.C., Rybicki B.A., Peterson E.L., Richardson R.J. (1998). The risk of Parkinson’s disease with exposure to pesticides, farming, well water, and rural living. Neurology.

[6] Nonnekes J., Post B., Tetrud J.W., Langston J.W., Bloem B.R. (2018). MPTP-induced parkinsonism: An historical case series. Lancet Neurol..

[7] Jankovic J., Tan E.K. (2020). Parkinson’s disease: Etiopathogenesis and treatment. J. Neurol. Neurosurg. Psychiatry.

[8] Ball N., Teo W.P., Chandra S., Chapman J. (2019). Parkinson’s Disease and the Environment. Front. Neurol..

[9] Lin T.K., Lin K.J., Lin K.L., Liou C.W., Chen S.D., Chuang Y.C., Wang P.W., Chuang J.H., Wang T.J. (2020). When Friendship Turns Sour: Effective Communication between Mitochondria and Intracellular Organelles in Parkinson’s Disease. Front. Cell Dev. Biol..

[10] Spinelli J.B., Haigis M.C. (2018). The multifaceted contributions of mitochondria to cellular metabolism. Nat. Cell Biol..

[11] Park J.S., Davis R.L., Sue C.M. (2018). Mitochondrial Dysfunction in Parkinson’s Disease: New Mechanistic Insights and Therapeutic Perspectives. Curr. Neurol. Neurosci. Rep..

[12] Larsen S.B., Hanss Z., Kruger R. (2018). The genetic architecture of mitochondrial dysfunction in Parkinson’s disease. Cell Tissue Res..

[13] Lesage S., Drouet V., Majounie E., Deramecourt V., Jacoupy M., Nicolas A., Cormier-Dequaire F., Hassoun S.M., Pujol C., Ciura S. (2016). Loss of VPS13C Function in Autosomal-Recessive Parkinsonism Causes Mitochondrial Dysfunction and Increases PINK1/Parkin-Dependent Mitophagy. Am. J. Hum. Genet..

[14] Nguyen M., Wong Y.C., Ysselstein D., Severino A., Krainc D. (2019). Synaptic, Mitochondrial, and Lysosomal Dysfunction in Parkinson’s Disease. Trends Neurosci..

[15] Smolders S., Van Broeckhoven C. (2020). Genetic perspective on the synergistic connection between vesicular transport, lysosomal and mitochondrial pathways associated with Parkinson’s disease pathogenesis. Acta Neuropathol. Commun..

[16] Lin K.J., Lin K.L., Chen S.D., Liou C.W., Chuang Y.C., Lin H.Y., Lin T.K. (2019). The Overcrowded Crossroads: Mitochondria, Alpha-Synuclein, and the Endo-Lysosomal System Interaction in Parkinson’s Disease. Int. J. Mol. Sci..

[17] Kinghorn K.J., Castillo-Quan J.I., Bartolome F., Angelova P.R., Li L., Pope S., Cochemé H.M., Khan S., Asghari S., Bhatia K.P. (2015). Loss of PLA2G6 leads to elevated mitochondrial lipid peroxidation and mitochondrial dysfunction. Brain.

[18] Billingsley K.J., Barbosa I.A., Bandrés-Ciga S., Quinn J.P., Bubb V.J., Deshpande C., Botia J.A., Reynolds R.H., Zhang D., Simpson M.A. (2019). Mitochondria function associated genes contribute to Parkinson’s Disease risk and later age at onset. NPJ Parkinson’s Dis..

[19] O’Regan G., deSouza R.M., Balestrino R., Schapira A.H. (2017). Glucocerebrosidase Mutations in Parkinson Disease. J. Parkinson’s Dis..

[20] Yue X., Li H., Yan H., Zhang P., Chang L., Li T. (2016). Risk of Parkinson Disease in Diabetes Mellitus: An Updated Meta-Analysis of Population-Based Cohort Studies. Medicine.

[21] De Pablo-Fernandez E., Goldacre R., Pakpoor J., Noyce A.J., Warner T.T. (2018). Association between diabetes and subsequent Parkinson disease: A record-linkage cohort study. Neurology.

[22] Yang Y.-W., Hsieh T.-F., Li C.-I., Liu C.-S., Lin W.-Y., Chiang J.-H., Li T.-C., Lin C.-C. (2017). Increased risk of Parkinson disease with diabetes mellitus in a population-based study. Medicine.

[23] Renaud J., Bassareo V., Beaulieu J., Pinna A., Schlich M., Lavoie C., Murtas D., Simola N., Martinoli M.-G. (2018). Dopaminergic neurodegeneration in a rat model of long-term hyperglycemia: Preferential degeneration of the nigrostriatal motor pathway. Neurobiol. Aging.

[24] Sandyk R. (1993). The relationship between diabetes mellitus and Parkinson’s disease. Int. J. Neurosci..

[25] Cheong J.L.Y., de Pablo-Fernandez E., Foltynie T., Noyce A.J. (2020). The Association between Type 2 Diabetes Mellitus and Parkinson’s Disease. J. Parkinson’s Dis..

[26] Olgar Y., Tuncay E., Degirmenci S., Billur D., Dhingra R., Kirshenbaum L., Turan B. (2020). Ageing-associated increase in SGLT2 disrupts mitochondrial/sarcoplasmic reticulum Ca(2+) homeostasis and promotes cardiac dysfunction. J. Cell. Mol. Med..

[27] Bray J.J.H., Foster-Davies H., Stephens J.W. (2020). A systematic review examining the effects of sodium-glucose cotransporter-2 inhibitors (SGLT2is) on biomarkers of inflammation and oxidative stress. Diabetes Res. Clin. Pract..

[28] Steven S., Oelze M., Hanf A., Kröller-Schön S., Kashani F., Roohani S., Welschof P., Kopp M., Gödtel-Armbrust U., Xia N. (2017). The SGLT2 inhibitor empagliflozin improves the primary diabetic complications in ZDF rats. Redox Biol..

[29] Liu L., Ni Y.Q., Zhan J.K., Liu Y.S. (2021). The Role of SGLT2 Inhibitors in Vascular Aging. Aging Dis..

[30] Lee W.-C., Chau Y.-Y., Ng H.-Y., Chen C.-H., Wang P.-W., Liou C.-W., Lin T.-K., Chen J.-B. (2019). Empagliflozin Protects HK-2 Cells from High Glucose-Mediated Injuries via a Mitochondrial Mechanism. Cells.

[31] Kuszak A.J., Espey M.G., Falk M.J., Holmbeck M.A., Manfredi G., Shadel G.S., Vernon H.J., Zolkipli-Cunningham Z. (2018). Nutritional Interventions for Mitochondrial OXPHOS Deficiencies: Mechanisms and Model Systems. Annu. Rev. Pathol..

[32] Riley J.S., Tait S.W. (2020). Mitochondrial DNA in inflammation and immunity. EMBO Rep..

[33] Lyngsie G., Krumina L., Tunlid A., Persson P. (2018). Generation of hydroxyl radicals from reactions between a dimethoxyhydroquinone and iron oxide nanoparticles. Sci. Rep..

[34] Pham A.N., Xing G., Miller C.J., Waite T.D. (2013). Fenton-like copper redox chemistry revisited: Hydrogen peroxide and superoxide mediation of copper-catalyzed oxidant production. J. Catal..

[35] Murphy M.P. (2009). How mitochondria produce reactive oxygen species. Biochem. J..

[36] Zelko I.N., Mariani T.J., Folz R.J. (2002). Superoxide dismutase multigene family: A comparison of the CuZn-SOD (SOD1), Mn-SOD (SOD2), and EC-SOD (SOD3) gene structures, evolution, and expression. Free Radic. Biol. Med..

[37] Lin H.Y., Chuang J.H., Wang P.W., Lin T.K., Wu M.T., Hsu W.M., Chuang H.C. (2020). 5-aza-2’-Deoxycytidine Induces a RIG-I-Related Innate Immune Response by Modulating Mitochondria Stress in Neuroblastoma. Cells.

[38] Han W., Fessel J.P., Sherrill T., Kocurek E.G., Yull F.E., Blackwell T.S. (2020). Enhanced Expression of Catalase in Mitochondria Modulates NF-κB-Dependent Lung Inflammation through Alteration of Metabolic Activity in Macrophages. J. Immunol..

[39] Lin T.K., Man M.Q., Abuabara K., Wakefield J.S., Sheu H.M., Tsai J.C., Lee C.H., Elias P.M. (2019). By protecting against cutaneous inflammation, epidermal pigmentation provided an additional advantage for ancestral humans. Evol. Appl..

[40] Eisner V., Picard M., Hajnóczky G. (2018). Mitochondrial dynamics in adaptive and maladaptive cellular stress responses. Nat. Cell Biol..

[41] Hoitzing H., Johnston I.G., Jones N.S. (2015). What is the function of mitochondrial networks? A theoretical assessment of hypotheses and proposal for future research. BioEssays.

[42] Chen H., Vermulst M., Wang Y.E., Chomyn A., Prolla T.A., McCaffery J.M., Chan D.C. (2010). Mitochondrial fusion is required for mtDNA stability in skeletal muscle and tolerance of mtDNA mutations. Cell.

[43] Alexander C., Votruba M., Pesch U.E., Thiselton D.L., Mayer S., Moore A., Rodriguez M., Kellner U., Leo-Kottler B., Auburger G. (2000). OPA1, encoding a dynamin-related GTPase, is mutated in autosomal dominant optic atrophy linked to chromosome 3q28. Nat. Genet..

[44] Tilokani L., Nagashima S., Paupe V., Prudent J. (2018). Mitochondrial dynamics: Overview of molecular mechanisms. Essays Biochem..

[45] Fonseca T.B., Sanchez-Guerrero A., Milosevic I., Raimundo N. (2019). Mitochondrial fission requires DRP1 but not dynamins. Nature.

[46] Modi S., López-Doménech G., Halff E.F., Covill-Cooke C., Ivankovic D., Melandri D., Arancibia-Cárcamo I.L., Burden J.J., Lowe A.R., Kittler J.T. (2019). Miro clusters regulate ER-mitochondria contact sites and link cristae organization to the mitochondrial transport machinery. Nat. Commun..

[47] Giorgi C., Marchi S., Pinton P. (2018). The machineries, regulation and cellular functions of mitochondrial calcium. Nat. Rev. Mol. Cell Biol..

[48] Belosludtsev K.N., Belosludtseva N.V., Dubinin M.V. (2020). Diabetes Mellitus, Mitochondrial Dysfunction and Ca2+-Dependent Permeability Transition Pore. Int. J. Mol. Sci..

[49] Andreasson C., Ott M., Buttner S. (2019). Mitochondria orchestrate proteostatic and metabolic stress responses. EMBO Rep..

[50] Shpilka T., Haynes C.M. (2018). The mitochondrial UPR: Mechanisms, physiological functions and implications in ageing. Nat. Rev. Mol. Cell Biol..

[51] Suh D.H., Kim M.-K., Kim H.S., Chung H.H., Song Y.S. (2013). Mitochondrial permeability transition pore as a selective target for anti-cancer therapy. Front. Oncol..

[52] Rasheed M.Z., Tabassum H., Parvez S. (2017). Mitochondrial permeability transition pore: A promising target for the treatment of Parkinson’s disease. Protoplasma.

[53] Shevtsova E.F., Maltsev A.V., Vinogradova D.V., Shevtsov P.N., Bachurin S.O. (2021). Mitochondria as a promising target for developing novel agents for treating Alzheimer’s disease. Med. Res. Rev..

[54] Briston T., Roberts M., Lewis S., Powney B., Staddon J.M., Szabadkai G., Duchen M.R. (2017). Mitochondrial permeability transition pore: Sensitivity to opening and mechanistic dependence on substrate availability. Sci. Rep..

[55] Wacquier B., Combettes L., Dupont G. (2020). Dual dynamics of mitochondrial permeability transition pore opening. Sci. Rep..

[56] Jiang M., Qi L., Li L., Li Y. (2020). The caspase-3/GSDME signal pathway as a switch between apoptosis and pyroptosis in cancer. Cell Death Discov..

[57] Lee Y., Chiou Y.J., Hung C.F., Chang Y.Y., Chen Y.F., Lin T.K., Wang L.J. (2021). A dyadic study of psychological well-being of individuals with Parkinson’s disease and their caregivers. Sci. Rep..

[58] Singh R., Letai A., Sarosiek K. (2019). Regulation of apoptosis in health and disease: The balancing act of BCL-2 family proteins. Nat. Rev. Mol. Cell Biol..

[59] Saeedi P., Petersohn I., Salpea P., Malanda B., Karuranga S., Unwin N., Colagiuri S., Guariguata L., Motala A.A., Ogurtsova K. (2019). Global and regional diabetes prevalence estimates for 2019 and projections for 2030 and 2045: Results from the International Diabetes Federation Diabetes Atlas, 9th edition. Diabetes Res. Clin. Pract..

[60] Schwab R.S. (1960). Progression and Prognosis in Parkinson’s Disease. J. Nerv. Ment. Dis..

[61] Hu G., Jousilahti P., Bidel S., Antikainen R., Tuomilehto J. (2007). Type 2 diabetes and the risk of Parkinson’s disease. Diabetes Care.

[62] Schernhammer E., Hansen J., Rugbjerg K., Wermuth L., Ritz B. (2011). Diabetes and the risk of developing Parkinson’s disease in Denmark. Diabetes Care.

[63] Xu Q., Park Y., Huang X., Hollenbeck A., Blair A., Schatzkin A., Chen H. (2011). Diabetes and risk of Parkinson’s disease. Diabetes Care.

[64] Pagano G., Polychronis S., Wilson H., Giordano B., Ferrara N., Niccolini F., Politis M. (2018). Diabetes mellitus and Parkinson disease. Neurology.

[65] Ong M., Foo H., Chander R.J., Wen M.-C., Au W.L., Sitoh Y.Y., Tan L., Kandiah N. (2017). Influence of diabetes mellitus on longitudinal atrophy and cognition in Parkinson’s disease. J. Neurol. Sci..

[66] Marques A., Dutheil F., Durand E., Rieu I., Mulliez A., Fantini M.L., Boirie Y., Durif F. (2018). Glucose dysregulation in Parkinson’s disease: Too much glucose or not enough insulin?. Parkinsonism Relat. Disord..

[67] Duarte A.I., Moreira P.I., Oliveira C.R. (2012). Insulin in central nervous system: More than just a peripheral hormone. J. Aging Res..

[68] Blázquez E., Velázquez E., Hurtado-Carneiro V., Ruiz-Albusac J.M. (2014). Insulin in the Brain: Its Pathophysiological Implications for States Related with Central Insulin Resistance, Type 2 Diabetes and Alzheimer’s Disease. Front. Endocrinol..

[69] Young W.S. (1986). Periventricular hypothalamic cells in the rat brain contain insulin mRNA. Neuropeptides.

[70] Frölich L., Blum-Degen D., Bernstein H.G., Engelsberger S., Humrich J., Laufer S., Muschner D., Thalheimer A., Türk A., Hoyer S. (1998). Brain insulin and insulin receptors in aging and sporadic Alzheimer’s disease. J. Neural Transm..

[71] Dorn A., Rinne A., Bernstein H.G., Hahn H.J., Ziegler M. (1983). Insulin and C-peptide in human brain neurons (insulin/C-peptide/brain peptides/immunohistochemistry/radioimmunoassay). J. Fur Hirnforsch..

[72] Pagotto U. (2009). Where does insulin resistance start? The brain. Diabetes Care.

[73] Könner A.C., Brüning J.C. (2011). Toll-like receptors: Linking inflammation to metabolism. Trends Endocrinol. Metab..

[74] Boden G. (2006). Fatty acid—induced inflammation and insulin resistance in skeletal muscle and liver. Curr. Diabetes Rep..

[75] Sripetchwandee J., Chattipakorn N., Chattipakorn S.C. (2018). Links between Obesity-Induced Brain Insulin Resistance, Brain Mitochondrial Dysfunction, and Dementia. Front. Endocrinol..

[76] Fiory F., Perruolo G., Cimmino I., Cabaro S., Pignalosa F.C., Miele C., Beguinot F., Formisano P., Oriente F. (2019). The Relevance of Insulin Action in the Dopaminergic System. Front. Neurosci..

[77] Takahashi M., Yamada T., Tooyama I., Moroo I., Kimura H., Yamamoto T., Okada H. (1996). Insulin receptor mRNA in the substantia nigra in Parkinson’s disease. Neurosci. Lett..

[78] Moloney A.M., Griffin R.J., Timmons S., O’Connor R., Ravid R., O’Neill C. (2010). Defects in IGF-1 receptor, insulin receptor and IRS-1/2 in Alzheimer’s disease indicate possible resistance to IGF-1 and insulin signalling. Neurobiol. Aging.

[79] Chou S.-Y., Chan L., Chung C.-C., Chiu J.-Y., Hsieh Y.-C., Hong C.-T. (2020). Altered Insulin Receptor Substrate 1 Phosphorylation in Blood Neuron-Derived Extracellular Vesicles from Patients with Parkinson’s Disease. Front. Cell Dev. Biol..

[80] Gao S., Duan C., Gao G., Wang X., Yang H. (2015). Alpha-synuclein overexpression negatively regulates insulin receptor substrate 1 by activating mTORC1/S6K1 signaling. Int. J. Biochem. Cell Biol..

[81] Hölscher C. (2020). Brain insulin resistance: Role in neurodegenerative disease and potential for targeting. Expert Opin. Investig. Drugs.

[82] Morris J.K., Vidoni E.D., Perea R.D., Rada R., Johnson D.K., Lyons K., Pahwa R., Burns J.M., Honea R.A. (2014). Insulin resistance and gray matter volume in neurodegenerative disease. Neuroscience.

[83] Hogg E., Athreya K., Basile C., Tan E.E., Kaminski J., Tagliati M. (2018). High Prevalence of Undiagnosed Insulin Resistance in Non-Diabetic Subjects with Parkinson’s Disease. J. Parkinson’s Dis..

[84] Markaki I., Ntetsika T., Sorjonen K., Svenningsson P., BioPark Study Group (2021). Euglycemia Indicates Favorable Motor Outcome in Parkinson’s Disease. Mov. Disord..

[85] Dubowitz N., Xue W., Long Q., Ownby J.G., Olson D.E., Barb D., Rhee M.K., Mohan A.V., Watson-Williams P.I., Jackson S.L. (2014). Aging is associated with increased HbA1c levels, independently of glucose levels and insulin resistance, and also with decreased HbA1c diagnostic specificity. Diabet. Med..

[86] Brunerova L., Potockova J., Horacek J., Suchy J., Andel M. (2013). Central Dopaminergic Activity Influences Metabolic Parameters in Healthy Men. Neuroendocrinology.

[87] Glei D.A., Goldman N., Lin Y.H., Weinstein M. (2011). Age-Related Changes in Biomarkers: Longitudinal Data from a Population-Based Sample. Res. Aging.

[88] De Mello N.P., Orellana A.M., Mazucanti C.H., de Morais Lima G., Scavone C., Kawamoto E.M. (2019). Insulin and Autophagy in Neurodegeneration. Front. Neurosci..

[89] Prasun P. (2020). Mitochondrial dysfunction in metabolic syndrome. Biochim. Biophys. Acta.

[90] Lin K.L., Chen S.D., Lin K.J., Liou C.W., Chuang Y.C., Wang P.W., Chuang J.H., Lin T.K. (2021). Quality Matters? The Involvement of Mitochondrial Quality Control in Cardiovascular Disease. Front. Cell Dev. Biol..

[91] Yang X., Cheng B. (2010). Neuroprotective and Anti-inflammatory Activities of Ketogenic Diet on MPTP-induced Neurotoxicity. J. Mol. Neurosci..

[92] Cheng B., Yang X., An L., Gao B., Liu X., Liu S. (2009). Ketogenic diet protects dopaminergic neurons against 6-OHDA neurotoxicity via up-regulating glutathione in a rat model of Parkinson’s disease. Brain Res..

[93] Okubo H., Miyake Y., Sasaki S., Murakami K., Tanaka K., Fukushima W., Kiyohara C., Tsuboi Y., Yamada T., Oeda T. (2012). Dietary patterns and risk of Parkinson’s disease: A case-control study in Japan. Eur. J. Neurol..

[94] Murakami K., Miyake Y., Sasaki S., Tanaka K., Fukushima W., Kiyohara C., Tsuboi Y., Yamada T., Oeda T., Miki T. (2010). Dietary glycemic index is inversely associated with the risk of Parkinson’s disease: A case–control study in Japan. Nutrition.

[95] Ighodaro O.M. (2018). Molecular pathways associated with oxidative stress in diabetes mellitus. Biomed. Pharmacother..

[96] Meza C.A., La Favor J.D., Kim D.H., Hickner R.C. (2019). Endothelial Dysfunction: Is There a Hyperglycemia-Induced Imbalance of NOX and NOS?. Int. J. Mol. Sci..

[97] Circu M.L., Aw T.Y. (2010). Reactive oxygen species, cellular redox systems, and apoptosis. Free Radic. Biol. Med..

[98] Nishikawa T., Edelstein D., Du X.L., Yamagishi S.-I., Matsumura T., Kaneda Y., Yorek M.A., Beebe D., Oates P.J., Hammes H.-P. (2000). Normalizing mitochondrial superoxide production blocks three pathways of hyperglycaemic damage. Nature.

[99] Ly L.D., Xu S., Choi S.-K., Ha C.-M., Thoudam T., Cha S.-K., Wiederkehr A., Wollheim C.B., Lee I.-K., Park K.-S. (2017). Oxidative stress and calcium dysregulation by palmitate in type 2 diabetes. Exp. Mol. Med..

[100] Ma Z.A., Zhao Z., Turk J. (2012). Mitochondrial Dysfunction and *β*-Cell Failure in Type 2 Diabetes Mellitus. Exp. Diabetes Res..

[101] Esteghamati A., Eskandari D., Mirmiranpour H., Noshad S., Mousavizadeh M., Hedayati M., Nakhjavani M. (2013). Effects of metformin on markers of oxidative stress and antioxidant reserve in patients with newly diagnosed type 2 diabetes: A randomized clinical trial. Clin. Nutr..

[102] Vial G., Detaille D., Guigas B. (2019). Role of Mitochondria in the Mechanism(s) of Action of Metformin. Front. Endocrinol..

[103] Ma J., Coarfa C., Qin X., Bonnen P.E., Milosavljevic A., Versalovic J., Aagaard K. (2014). mtDNA haplogroup and single nucleotide polymorphisms structure human microbiome communities. BMC Genom..

[104] Cavalcante G.C., Magalhaes L., Ribeiro-Dos-Santos A., Vidal A.F. (2020). Mitochondrial Epigenetics: Non-Coding RNAs as a Novel Layer of Complexity. Int. J. Mol. Sci..

[105] Cha M.-Y., Kim D.K., Mook-Jung I. (2015). The role of mitochondrial DNA mutation on neurodegenerative diseases. Exp. Mol. Med..

[106] Kumari T., Vachher M., Bansal S., Bamezai R.N.K., Kumar B. (2018). Meta-analysis of mitochondrial T16189C polymorphism for cancer and Type 2 diabetes risk. Clin. Chim. Acta.

[107] Liou C.W., Lin T.K., Huang F.M., Chen T.L., Lee C.F., Chuang Y.C., Tan T.Y., Chang K.C., Wei Y.H. (2004). Association of the mitochondrial DNA 16189 T to C variant with lacunar cerebral infarction: Evidence from a hospital-based case-control study. Ann. N. Y. Acad. Sci..

[108] Chen Y.N., Liou C.W., Huang C.C., Lin T.K., Wei Y.H. (2004). Maternally inherited diabetes and deafness (MIDD) syndrome: A clinical and molecular genetic study of a Taiwanese family. Chang Gung Med. J..

[109] Liou C.W., Huang C.C., Lee C.F., Lin T.K., Wei Y.H. (2003). Low antioxidant content and mutation load in mitochondrial DNA A3243G mutation-related diabetes mellitus. J. Med. Assoc..

[110] Liou C.W., Huang C.C., Lin T.K., Tsai J.L., Wei Y.H. (2000). Correction of pancreatic beta-cell dysfunction with coenzyme Q(10) in a patient with mitochondrial encephalomyopathy, lactic acidosis and stroke-like episodes syndrome and diabetes mellitus. Eur. Neurol..

[111] McDonnell M.T., Schaefer A.M., Blakely E.L., McFarland R., Chinnery P.F., Turnbull D.M., Taylor R.W. (2004). Noninvasive diagnosis of the 3243A>G mitochondrial DNA mutation using urinary epithelial cells. Eur. J. Hum. Genet..

[112] Ikeda T., Osaka H., Shimbo H., Tajika M., Yamazaki M., Ueda A., Murayama K., Yamagata T. (2018). Mitochondrial DNA 3243A>T mutation in a patient with MELAS syndrome. Hum. Genome Var..

[113] Liou C.W., Lin T.K., Chen J.B., Tiao M.M., Weng S.W., Chen S.D., Chuang Y.C., Chuang J.H., Wang P.W. (2010). Association between a common mitochondrial DNA D-loop polycytosine variant and alteration of mitochondrial copy number in human peripheral blood cells. J. Med. Genet..

[114] Wang P.W., Lin T.K., Weng S.W., Liou C.W. (2009). Mitochondrial DNA variants in the pathogenesis of type 2 diabetes—Relevance of asian population studies. Rev. Diabet. Stud..

[115] Tiao M.M., Lin T.K., Liou C.W., Wang P.W., Chen J.B., Kuo F.Y., Huang C.C., Chou Y.M., Chuang J.H. (2009). Early transcriptional deregulation of hepatic mitochondrial biogenesis and its consequent effects on murine cholestatic liver injury. Apoptosis.

[116] Liou C.W., Lin T.K., Huei Weng H., Lee C.F., Chen T.L., Wei Y.H., Chen S.D., Chuang Y.C., Weng S.W., Wang P.W. (2007). A common mitochondrial DNA variant and increased body mass index as associated factors for development of type 2 diabetes: Additive effects of genetic and environmental factors. J. Clin. Endocrinol. Metab..

[117] Lin T.K., Chen S.D., Wang P.W., Wei Y.H., Lee C.F., Chen T.L., Chuang Y.C., Tan T.Y., Chang K.C., Liou C.W. (2005). Increased oxidative damage with altered antioxidative status in type 2 diabetic patients harboring the 16189 T to C variant of mitochondrial DNA. Ann. N. Y. Acad. Sci..

[118] Van Oven M., Kayser M. (2009). Updated comprehensive phylogenetic tree of global human mitochondrial DNA variation. Hum. Mutat..

[119] Tsai M.H., Kuo C.W., Lin T.K., Ho C.J., Wang P.W., Chuang J.H., Liou C.W. (2020). Ischemic Stroke Risk Associated with Mitochondrial Haplogroup F in the Asian Population. Cells.

[120] Liou C.W., Chuang J.H., Chen J.B., Tiao M.M., Wang P.W., Huang S.T., Huang T.L., Lee W.C., Weng S.W., Huang P.H. (2016). Mitochondrial DNA variants as genetic risk factors for Parkinson disease. Eur. J. Neurol..

[121] Wu H.M., Li T., Wang Z.F., Huang S.S., Shao Z.Q., Wang K., Zhong H.Q., Chen S.F., Zhang X., Zhu J.H. (2018). Mitochondrial DNA variants modulate genetic susceptibility to Parkinson’s disease in Han Chinese. Neurobiol. Dis..

[122] Aviles-Olmos I., Limousin P., Lees A., Foltynie T. (2013). Parkinson’s disease, insulin resistance and novel agents of neuroprotection. Brain.

[123] Wang X., Zeng F., Jin W.-S., Zhu C., Wang Q.-H., Bu X.-L., Luo H.-B., Zou H.-Q., Pu J., Zhou Z.-H. (2017). Comorbidity burden of patients with Parkinson’s disease and Parkinsonism between 2003 and 2012: A multicentre, nationwide, retrospective study in China. Sci. Rep..

[124] Sergi D., Renaud J., Simola N., Martinoli M.G. (2019). Diabetes, a Contemporary Risk for Parkinson’s Disease: Epidemiological and Cellular Evidences. Front. Aging Neurosci..

[125] Wahlqvist M.L., Lee M.S., Hsu C.C., Chuang S.Y., Lee J.T., Tsai H.N. (2012). Metformin-inclusive sulfonylurea therapy reduces the risk of Parkinson’s disease occurring with Type 2 diabetes in a Taiwanese population cohort. Parkinsonism Relat. Disord..

[126] Paudel Y.N., Angelopoulou E., Piperi C., Shaikh M.F., Othman I. (2020). Emerging neuroprotective effect of metformin in Parkinson’s disease: A molecular crosstalk. Pharmacol. Res..

[127] Mor D.E., Sohrabi S., Kaletsky R., Keyes W., Tartici A., Kalia V., Miller G.W., Murphy C.T. (2020). Metformin rescues Parkinson’s disease phenotypes caused by hyperactive mitochondria. Proc. Natl. Acad. Sci. USA.

[128] Rotermund C., Machetanz G., Fitzgerald J.C. (2018). The Therapeutic Potential of Metformin in Neurodegenerative Diseases. Front. Endocrinol..

[129] Carta A.R., Frau L., Pisanu A., Wardas J., Spiga S., Carboni E. (2011). Rosiglitazone decreases peroxisome proliferator receptor-γ levels in microglia and inhibits TNF-α production: New evidences on neuroprotection in a progressive Parkinson’s disease model. Neuroscience.

[130] Sadeghian M., Marinova-Mutafchieva L., Broom L., Davis J.B., Virley D., Medhurst A.D., Dexter D.T. (2012). Full and partial peroxisome proliferation-activated receptor-gamma agonists, but not delta agonist, rescue of dopaminergic neurons in the 6-OHDA parkinsonian model is associated with inhibition of microglial activation and MMP expression. J. Neuroimmunol..

[131] Brauer R., Bhaskaran K., Chaturvedi N., Dexter D.T., Smeeth L., Douglas I. (2015). Glitazone Treatment and Incidence of Parkinson’s Disease among People with Diabetes: A Retrospective Cohort Study. PLoS Med..

[132] Zhu Y., Pu J., Chen Y., Zhang B. (2019). Decreased risk of Parkinson’s disease in diabetic patients with thiazolidinediones therapy: An exploratory meta-analysis. PLoS ONE.

[133] Athauda D., Maclagan K., Skene S.S., Bajwa-Joseph M., Letchford D., Chowdhury K., Hibbert S., Budnik N., Zampedri L., Dickson J. (2017). Exenatide once weekly versus placebo in Parkinson’s disease: A randomised, double-blind, placebo-controlled trial. Lancet.

[134] Athauda D., Foltynie T. (2016). The glucagon-like peptide 1 (GLP) receptor as a therapeutic target in Parkinson’s disease: Mechanisms of action. Drug Discov. Today.

[135] Athauda D., Foltynie T. (2016). Insulin resistance and Parkinson’s disease: A new target for disease modification?. Prog. Neurobiol..

[136] Grieco M., Giorgi A., Gentile M.C., d’Erme M., Morano S., Maras B., Filardi T. (2019). Glucagon-Like Peptide-1: A Focus on Neurodegenerative Diseases. Front. Neurosci..

[137] Lin T.-K., Lin K.-J., Lin H.-Y., Lin K.-L., Lan M.-Y., Wang P.-W., Wang T.-J., Wang F.-S., Tsai P.-C., Liou C.-W. (2021). Glucagon-Like Peptide-1 Receptor Agonist Ameliorates 1-Methyl-4-Phenyl-1,2,3,6-Tetrahydropyridine (MPTP) Neurotoxicity Through Enhancing Mitophagy Flux and Reducing α-Synuclein and Oxidative Stress. Front. Mol. Neurosci..

[138] Badawi G.A., Abd El Fattah M.A., Zaki H.F., El Sayed M.I. (2019). Sitagliptin and Liraglutide Modulate L-dopa Effect and Attenuate Dyskinetic Movements in Rotenone-Lesioned Rats. Neurotox. Res..

[139] Jeong S.H., Chung S.J., Yoo H.S., Hong N., Jung J.H., Baik K., Lee Y.H., Sohn Y.H., Lee P.H. (2021). Beneficial effects of dipeptidyl peptidase-4 inhibitors in diabetic Parkinson’s disease. Brain.

[140] Brauer R., Wei L., Ma T., Athauda D., Girges C., Vijiaratnam N., Auld G., Whittlesea C., Wong I., Foltynie T. (2020). Diabetes medications and risk of Parkinson’s disease: A cohort study of patients with diabetes. Brain.

[141] Cowie M.R., Fisher M. (2020). SGLT2 inhibitors: Mechanisms of cardiovascular benefit beyond glycaemic control. Nat. Rev. Cardiol..

[142] Chao E.C., Henry R.R. (2010). SGLT2 inhibition—A novel strategy for diabetes treatment. Nat. Rev. Drug Discov..

[143] Dekkers C.C.J., Sjöström C.D., Greasley P.J., Cain V., Boulton D.W., Heerspink H.J.L. (2019). Effects of the sodium-glucose co-transporter-2 inhibitor dapagliflozin on estimated plasma volume in patients with type 2 diabetes. Diabetes Obes. Metab..

[144] Zinman B., Wanner C., Lachin J.M., Fitchett D., Bluhmki E., Hantel S., Mattheus M., Devins T., Johansen O.E., Woerle H.J. (2015). Empagliflozin, Cardiovascular Outcomes, and Mortality in Type 2 Diabetes. N. Engl. J. Med..

[145] Neal B., Perkovic V., Mahaffey K.W., de Zeeuw D., Fulcher G., Erondu N., Shaw W., Law G., Desai M., Matthews D.R. (2017). Canagliflozin and Cardiovascular and Renal Events in Type 2 Diabetes. N. Engl. J. Med..

[146] Wiviott S.D., Raz I., Bonaca M.P., Mosenzon O., Kato E.T., Cahn A., Silverman M.G., Zelniker T.A., Kuder J.F., Murphy S.A. (2019). Dapagliflozin and Cardiovascular Outcomes in Type 2 Diabetes. N. Engl. J. Med..

[147] Kosiborod M., Cavender M.A., Fu A.Z., Wilding J.P., Khunti K., Holl R.W., Norhammar A., Birkeland K.I., Jørgensen M.E., Thuresson M. (2017). Lower Risk of Heart Failure and Death in Patients Initiated on Sodium-Glucose Cotransporter-2 Inhibitors Versus Other Glucose-Lowering Drugs: The CVD-REAL Study (Comparative Effectiveness of Cardiovascular Outcomes in New Users of Sodium-Glucose Cotransporter-2 Inhibitors). Circulation.

[148] McMurray J.J.V., Solomon S.D., Inzucchi S.E., Køber L., Kosiborod M.N., Martinez F.A., Ponikowski P., Sabatine M.S., Anand I.S., Bělohlávek J. (2019). Dapagliflozin in Patients with Heart Failure and Reduced Ejection Fraction. N. Engl. J. Med..

[149] Yaribeygi H., Atkin S.L., Butler A.E., Sahebkar A. (2019). Sodium-glucose cotransporter inhibitors and oxidative stress: An update. J. Cell. Physiol..

[150] Esterline R.L., Vaag A., Oscarsson J., Vora J. (2018). MECHANISMS IN ENDOCRINOLOGY: SGLT2 inhibitors: Clinical benefits by restoration of normal diurnal metabolism?. Eur. J. Endocrinol..

[151] Shah K., Desilva S., Abbruscato T. (2012). The role of glucose transporters in brain disease: Diabetes and Alzheimer’s Disease. Int. J. Mol. Sci..

[152] Camargo Maluf F., Feder D., Alves de Siqueira Carvalho A. (2019). Analysis of the Relationship between Type II Diabetes Mellitus and Parkinson’s Disease: A Systematic Review. Parkinson’s Dis..

[153] De Lazzari F., Bubacco L., Whitworth A.J., Bisaglia M. (2018). Superoxide Radical Dismutation as New Therapeutic Strategy in Parkinson’s Disease. Aging Dis..

[154] Maejima Y. (2020). SGLT2 Inhibitors Play a Salutary Role in Heart Failure via Modulation of the Mitochondrial Function. Front. Cardiovasc. Med..

[155] Lopaschuk G.D. (2016). Fatty Acid Oxidation and Its Relation with Insulin Resistance and Associated Disorders. Ann. Nutr. Metab..

[156] Tomita I., Kume S., Sugahara S., Osawa N., Yamahara K., Yasuda-Yamahara M., Takeda N., Chin-Kanasaki M., Kaneko T., Mayoux E. (2020). SGLT2 Inhibition Mediates Protection from Diabetic Kidney Disease by Promoting Ketone Body-Induced mTORC1 Inhibition. Cell Metab..

[157] Russell R.C., Yuan H.-X., Guan K.-L. (2014). Autophagy regulation by nutrient signaling. Cell Res..

[158] Bartolomé A., García-Aguilar A., Asahara S.I., Kido Y., Guillén C., Pajvani U.B., Benito M. (2017). MTORC1 Regulates both General Autophagy and Mitophagy Induction after Oxidative Phosphorylation Uncoupling. Mol. Cell. Biol..

[159] Tsai K.-F., Chen Y.-L., Chiou T.T.-Y., Chu T.-H., Li L.-C., Ng H.-Y., Lee W.-C., Lee C.-T. (2021). Emergence of SGLT2 Inhibitors as Powerful Antioxidants in Human Diseases. Antioxidants.

[160] Feng R., Dong L., Wang L., Xu Y., Lu H., Zhang J. (2019). Development of sodium glucose co-transporter 2 (SGLT2) inhibitors with novel structure by molecular docking and dynamics simulation. J. Mol. Modeling.

[161] Cai W., Jiang L., Xie Y., Liu Y., Liu W., Zhao G. (2015). Design of SGLT2 Inhibitors for the Treatment of Type 2 Diabetes: A History Driven by Biology to Chemistry. Med. Chem..

[162] Ehrenkranz J.R., Lewis N.G., Kahn C.R., Roth J. (2005). Phlorizin: A review. Diabetes/Metab. Res. Rev..

[163] Zielinska D., Laparra-Llopis J.M., Zielinski H., Szawara-Nowak D., Giménez-Bastida J.A. (2019). Role of Apple Phytochemicals, Phloretin and Phloridzin, in Modulating Processes Related to Intestinal Inflammation. Nutrients.

[164] Prabhakar P., Ahmed A.B., Chidambaram S. (2020). The Role of Phloridzin and its Possible Potential Therapeutic Effect on Parkinson’s Disease. Int. J. Nutr. Pharmacol. Neurol. Dis..

[165] Osorio H., Coronel I., Arellano A., Pacheco U., Bautista R., Franco M., Escalante B. (2012). Sodium-glucose cotransporter inhibition prevents oxidative stress in the kidney of diabetic rats. Oxidative Med. Cell. Longev..

[166] Bierhaus A., Fleming T., Stoyanov S., Leffler A., Babes A., Neacsu C., Sauer S.K., Eberhardt M., Schnölzer M., Lasitschka F. (2012). Methylglyoxal modification of Nav1.8 facilitates nociceptive neuron firing and causes hyperalgesia in diabetic neuropathy. Nat. Med..

[167] Oshima H., Miki T., Kuno A., Mizuno M., Sato T., Tanno M., Yano T., Nakata K., Kimura Y., Abe K. (2019). Empagliflozin, an SGLT2 Inhibitor, Reduced the Mortality Rate after Acute Myocardial Infarction with Modification of Cardiac Metabolomes and Antioxidants in Diabetic Rats. J. Pharmacol. Exp. Ther..

[168] Sun X., Han F., Lu Q., Li X., Ren D., Zhang J., Han Y., Xiang Y.K., Li J. (2020). Empagliflozin Ameliorates Obesity-Related Cardiac Dysfunction by Regulating Sestrin2-Mediated AMPK-mTOR Signaling and Redox Homeostasis in High-Fat Diet-Induced Obese Mice. Diabetes.

[169] Andreadou I., Efentakis P., Balafas E., Togliatto G., Davos C.H., Varela A., Dimitriou C.A., Nikolaou P.-E., Maratou E., Lambadiari V. (2017). Empagliflozin Limits Myocardial Infarction in Vivo and Cell Death in Vitro: Role of STAT3, Mitochondria, and Redox Aspects. Front. Physiol..

[170] Iannantuoni F., de Marañon M.A., Diaz-Morales N., Falcon R., Bañuls C., Abad-Jimenez Z., Victor V.M., Hernandez-Mijares A., Rovira-Llopis S. (2019). The SGLT2 Inhibitor Empagliflozin Ameliorates the Inflammatory Profile in Type 2 Diabetic Patients and Promotes an Antioxidant Response in Leukocytes. J. Clin. Med..

[171] Takagi S., Li J., Takagaki Y., Kitada M., Nitta K., Takasu T., Kanasaki K., Koya D. (2018). Ipragliflozin improves mitochondrial abnormalities in renal tubules induced by a high-fat diet. J. Diabetes Investig..

[172] Tahara A., Kurosaki E., Yokono M., Yamajuku D., Kihara R., Hayashizaki Y., Takasu T., Imamura M., Li Q., Tomiyama H. (2014). Effects of sodium-glucose cotransporter 2 selective inhibitor ipragliflozin on hyperglycaemia, oxidative stress, inflammation and liver injury in streptozotocin-induced type 1 diabetic rats. J. Pharm. Pharmacol..

[173] Lin T.K., Chen S.D., Lin K.J., Chuang Y.C. (2020). Seizure-Induced Oxidative Stress in Status Epilepticus: Is Antioxidant Beneficial?. Antioxidants.

[174] Yang X., Liu Q., Li Y., Tang Q., Wu T., Chen L., Pu S., Zhao Y., Zhang G., Huang C. (2020). The diabetes medication canagliflozin promotes mitochondrial remodelling of adipocyte via the AMPK-Sirt1-Pgc-1α signalling pathway. Adipocyte.

[175] Durak A., Olgar Y., Degirmenci S., Akkus E., Tuncay E., Turan B. (2018). A SGLT2 inhibitor dapagliflozin suppresses prolonged ventricular-repolarization through augmentation of mitochondrial function in insulin-resistant metabolic syndrome rats. Cardiovasc. Diabetol..

[176] Lin B., Koibuchi N., Hasegawa Y., Sueta D., Toyama K., Uekawa K., Ma M., Nakagawa T., Kusaka H., Kim-Mitsuyama S. (2014). Glycemic control with empagliflozin, a novel selective SGLT2 inhibitor, ameliorates cardiovascular injury and cognitive dysfunction in obese and type 2 diabetic mice. Cardiovasc. Diabetol..

[177] Hierro-Bujalance C., Infante-Garcia C., del Marco A., Herrera M., Carranza-Naval M.J., Suarez J., Alves-Martinez P., Lubian-Lopez S., Garcia-Alloza M. (2020). Empagliflozin reduces vascular damage and cognitive impairment in a mixed murine model of Alzheimer’s disease and type 2 diabetes. Alzheimer’s Res. Ther..

[178] Amin E.F., Rifaai R.A., Abdel-Latif R.G. (2020). Empagliflozin attenuates transient cerebral ischemia/reperfusion injury in hyperglycemic rats via repressing oxidative-inflammatory-apoptotic pathway. Fundam. Clin. Pharmacol..

[179] Arafa N.M.S., Ali E.H.A., Hassan M.K. (2017). Canagliflozin prevents scopolamine-induced memory impairment in rats: Comparison with galantamine hydrobromide action. Chem.-Biol. Interact..

[180] Sa-nguanmoo P., Tanajak P., Kerdphoo S., Jaiwongkam T., Pratchayasakul W., Chattipakorn N., Chattipakorn S.C. (2017). SGLT2-inhibitor and DPP-4 inhibitor improve brain function via attenuating mitochondrial dysfunction, insulin resistance, inflammation, and apoptosis in HFD-induced obese rats. Toxicol. Appl. Pharmacol..

[181] Arab H.H., Safar M.M., Shahin N.N. (2021). Targeting ROS-Dependent AKT/GSK-3β/NF-κB and DJ-1/Nrf2 Pathways by Dapagliflozin Attenuates Neuronal Injury and Motor Dysfunction in Rotenone-Induced Parkinson’s Disease Rat Model. ACS Chem. Neurosci..

[182] Erdogan M.A., Yusuf D., Christy J., Solmaz V., Erdogan A., Taskiran E., Erbas O. (2018). Highly selective SGLT2 inhibitor dapagliflozin reduces seizure activity in pentylenetetrazol-induced murine model of epilepsy. BMC Neurol..

[183] Wang H.J., Tyagi P., Lin T.K., Huang C.C., Lee W.C., Chancellor M.B., Chuang Y.C. (2021). Low Energy Shock Wave Therapy Attenuates Mitochondrial Dysfunction and Improves Bladder Function in HCl induced Cystitis in Rats. Biomed. J..

[184] Barreca D., Currò M., Bellocco E., Ficarra S., Laganà G., Tellone E., Laura Giunta M., Visalli G., Caccamo D., Galtieri A. (2017). Neuroprotective effects of phloretin and its glycosylated derivative on rotenone-induced toxicity in human SH-SY5Y neuronal-like cells. BioFactors.

[185] Hayden M.R., Grant D.G., Aroor A.R., DeMarco V.G. (2019). Empagliflozin Ameliorates Type 2 Diabetes-Induced Ultrastructural Remodeling of the Neurovascular Unit and Neuroglia in the Female db/db Mouse. Brain Sci..

[186] Abdel-latif R.G., Rifaai R.A., Amin E.F. (2020). Empagliflozin alleviates neuronal apoptosis induced by cerebral ischemia/reperfusion injury through HIF-1α/VEGF signaling pathway. Arch. Pharmacal Res..

